# The Intestinal Microbiome Primes Host Innate Immunity against Enteric Virus Systemic Infection through Type I Interferon

**DOI:** 10.1128/mBio.00366-21

**Published:** 2021-05-11

**Authors:** Xiao-Lian Yang, Gan Wang, Jin-Yan Xie, Han Li, Shu-Xian Chen, Wei Liu, Shu Jeffrey Zhu

**Affiliations:** a Key Laboratory of Animal Virology of Ministry of Agriculture, Center for Veterinary Sciences, Zhejiang University, Hangzhou, People’s Republic of China; b Department of Critical Care Medicine, Sir Run Run Shaw Hospital, Zhejiang University School of Medicine, Hangzhou, People’s Republic of China; c State Key Laboratory of Pathogen and Biosecurity, Beijing Institute of Microbiology and Epidemiology, Beijing, People’s Republic of China; Washington University School of Medicine; Washington University School of Medicine

**Keywords:** microbiota, *B. coccoides*, macrophage, type I interferon, enteric virus

## Abstract

Intestinal microbiomes are of vital importance in antagonizing systemic viral infection. However, very little literature has shown whether commensal bacteria play a crucial role in protecting against enteric virus systemic infection from the aspect of modulating host innate immunity. In the present study, we utilized an enteric virus, encephalomyocarditis virus (EMCV), to inoculate mice treated with phosphate-buffered saline (PBS) or given an antibiotic cocktail (Abx) orally or intraperitoneally to examine the impact of microbiota depletion on virulence and viral replication *in vivo*. Microbiota depletion exacerbated the mortality, neuropathogenesis, viremia, and viral burden in brains following EMCV infection. Furthermore, Abx-treated mice exhibited severely diminished mononuclear phagocyte activation and impaired type I interferon (IFN) production and expression of IFN-stimulated genes (ISG) in peripheral blood mononuclear cells (PBMC), spleens, and brains. With the help of fecal bacterial 16S rRNA sequencing of PBS- and Abx-treated mice, we identified a single commensal bacterium, Blautia coccoides, that can restore mononuclear phagocyte- and IFNAR (IFN-α/β receptor)-dependent type I IFN responses to restrict systemic enteric virus infection. These findings may provide insight into the development of novel therapeutics for preventing enteric virus infection or possibly alleviating clinical diseases by activating host systemic innate immune responses via respective probiotic treatment using *B. coccoides*.

## INTRODUCTION

Mammalian intestines are colonized by trillions of microorganisms, composed of bacteria, viruses, archaea, and fungi, collectively referred to as intestinal microbiota (1, 2). The widely diverse intestinal microbial communities have established an immensely complicated ecosystem that is vital in maintaining host homeostasis. Cumulative evidence strongly supports the view that the dynamic cross talk between the host and its indigenous commensal bacteria is fundamental for the development, induction, education, and tuning of the host immune system (3). As such, perturbation of the microbiota composition (termed “dysbiosis”) is linked to a myriad of metabolic and inflammatory diseases, both at intestinal mucosal sites and outside the gastrointestinal tract (2, 4, 5).

Over the past decade, rapid and extensive advances have shed light on the nature and extreme importance of intestinal microbes in regulating virus replication, transmission, and pathogenesis. Multiple studies utilizing enteric viruses, including poliovirus, retrovirus, and noroviruses, have demonstrated that the intestinal bacteria can promote viral infection (6–8). The stimulating mechanisms include direct facilitation of viral binding to target cells, virion stabilization, and indirect regulatory pathways of suppressing the mucosal immune responses (7, 9, 10). However, as every coin has two sides, the signals from the indigenous microbiota are shown to be essential to protect *Drosophila* from peroral enteric virus infection by priming antiviral innate immunity (11).

Other than exhibiting a regulatory effect at mucosal sites during enteric viral infection, the gut microbiota has been shown to be essential in limiting viral systemic infection by calibrating innate immune responses mediated by mononuclear phagocytes (12–14). A role for the commensal bacteria in regulating systemic type I interferon (IFN-I) has been described in these studies, in which impaired production of IFN-I and interferon-stimulated genes (ISGs) was observed in antibiotic (Abx)-treated or germfree (GF) mice infected with influenza A virus, murine cytomegalovirus, or Sendai virus, showing increased mortality and susceptibility (13, 15, 16). Recently, two compelling back-to-back research articles reported that plasmacytoid dendritic cells (pDCs) are the main cellular sources of microbiota-induced IFN-I. Schaupp and colleagues demonstrated that microbiota-driven IFN-I expression by pDCs primes conventional dendric cells (cDCs) to initiate immune responses following pathogen encounter (17). Meanwhile, Diamond’s group pointed out that IFN-I produced by microbiota-enabled pDCs prevents alphaviruses, Chikungunya virus (CHIKV) in particular, from infecting and disseminating in host blood monocytes (18). The latter study demonstrated that a single commensal bacterium, Clostridium scindens, can modulate prompt IFN-I responses through Toll-like receptor 7 (TLR7) and MyD88 signaling in pDCs via primary to secondary bile acid (BA) transformation (18). Although these studies provided important insights into the systemic effect of microbiota to limit viral infection, identification of unknown specific microbiome species that influence type I IFN antiviral responses is still lacking, and the molecular links between the gut microbiota and type I IFN-mediated innate immunity are only beginning to be elucidated.

Here, we describe the impact of the intestinal microbiome on host IFN-I-associated antiviral innate immunity using encephalomyocarditis virus (EMCV), a well-studied picornavirus that targets the central nervous system and is transmitted via the fecal-oral route. Depletion of the gut microbiota with an Abx cocktail via oral gavage exacerbated the mortality and neurological symptoms of EMCV infection in mice. EMCV infection of Abx-treated mice resulted in increased viral loads in blood and brain tissues. Coincidental with these results, the Abx-treated mice exhibited diminished innate immune responses reflected by impacted mononuclear phagocyte activation after EMCV infection, and this was correlated with diminished expression of ISGs in the peripheral and brain tissues. We performed bacterial 16S rRNA sequencing of the fecal samples collected from the Abx cocktail-treated and single Abx-treated mice to identify the possible commensal bacteria that may play important roles in priming IFN-I expression. Monocolonization of Abx-treated mice with *B. coccoides* enabled them to regain the capability of restricting EMCV systemic infection by activating mononuclear phagocytes through type I interferon expression. Thus, in the present study, we have identified a commensal bacterium that, as far as we know, has not been previously documented to be able to regulate type I IFN-mediated innate immunity in the context of enteric systemic virus infection.

## RESULTS

### Microbiota depletion alters the neuropathogenesis and mortality of EMCV infection.

**FIG 1:**
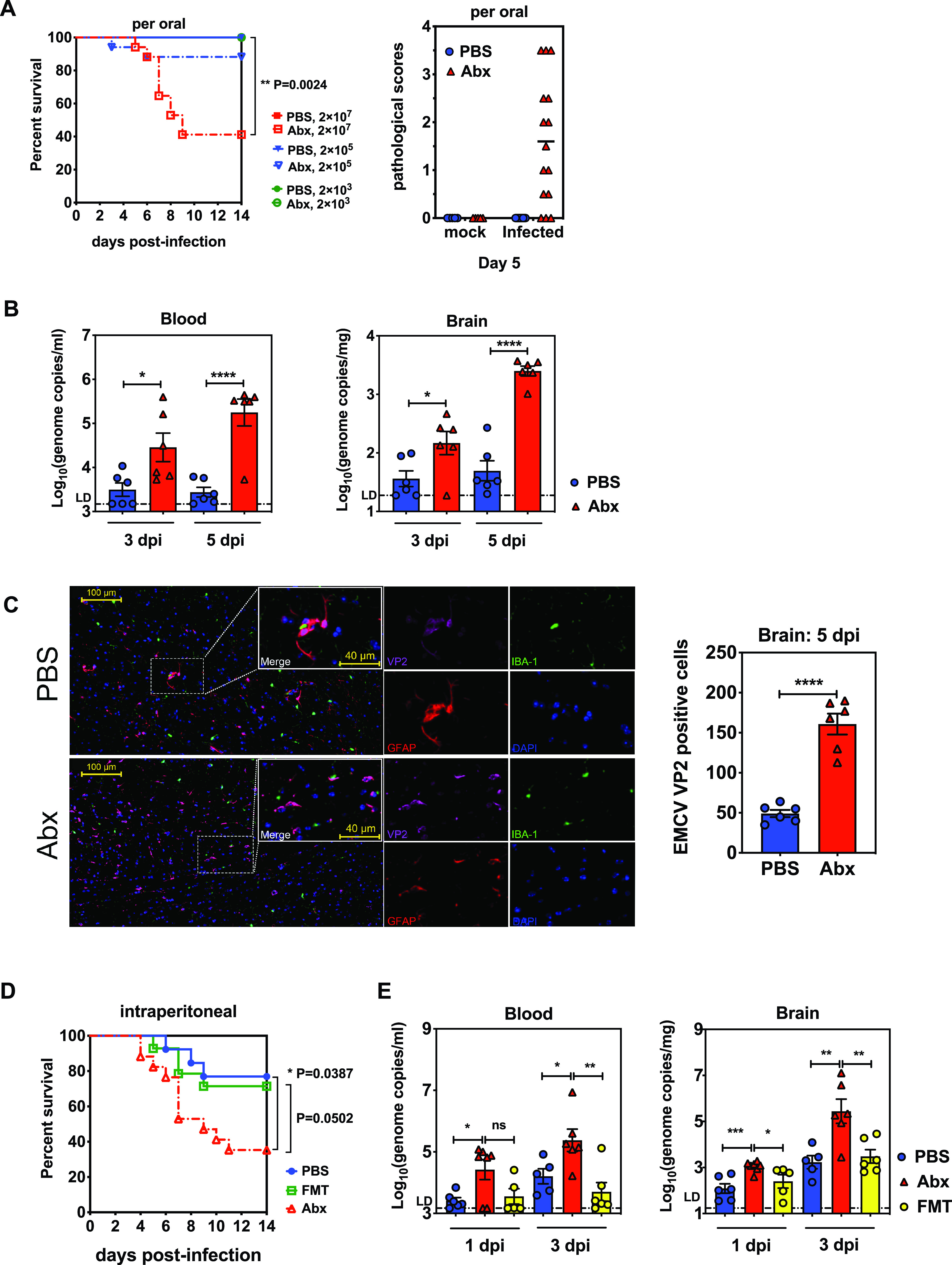
Microbiota depletion exacerbates EMCV pathogenesis and viral replication *in vivo*. (A) Groups of 6- to 8-week-old C57BL/6J mice were pretreated with PBS or Abx for 5 days, inoculated with different doses of EMCV orally, and observed for 14 days. Survival curves (*n* = 12 to 17) and pathological scores (*n* = 10 to 15) were documented. (B) Blood and brain samples of PBS- or Abx-treated mice inoculated with 2 × 10^7^ TCID_50_s of EMCV orally were harvested and tested for viral loads at 3 and 5 dpi by qPCR (*n* = 6). (C) IFA of brain sections from PBS- or Abx-treated mice at 5 dpi. EMCV capsid protein VP2 and cellular surface markers were double stained with the respective antibodies (representative images from 6 per group). (D) Groups of PBS-treated, Abx-treated, or Abx-treated FMT recipient mice (mice that received FMT from conventionally housed PBS-treated naive mice) were intraperitoneally inoculated with 200 TCID_50_s of EMCV for survival kinetics analysis (*n* = 13 to 17). (E) Viral titers of brain tissues collected from PBS-treated, Abx-treated, or FMT recipient mice at 1 and 3 dpi (same dose and route as for panel D; *n* = 5 or 6). Data are from two independent experiments. Broken lines indicate the limit of detection (LD). *, *P* < 0.05; **, *P* < 0.01; ***, *P* < 0.001; ****, *P* < 0.0001; ns, not significant.

Groups of wild-type C57BL/6J mice (referred to here as WT B6 mice) were subjected to oral gavage with a broad-spectrum Abx cocktail, composed of vancomycin (Van), neomycin (Neo), ampicillin (Amp), and metronidazole (Metro), consecutively for 5 days and infected orally with different doses of EMCV; the same Abx were added to the animals’ drinking water until the end of the experiments postinoculation (6, 19, 20). The Abx-treated mice and phosphate-buffered saline (PBS)-treated control animals were subsequently inoculated with different doses of EMCV in a 25-μl inoculum orally to mimic the route of natural infection. The clinical symptoms and mortality rates of different groups were observed and documented for 14 days postinfection (dpi). As shown in Fig. 1A, 60% of Abx-treated mice inoculated with high dose of 2 × 10^7^ 50% tissue culture infective doses (TCID_50_s) succumbed to infection by 9 dpi, only about 10% of Abx-treated mice succumbed at the dose of 2 × 10^5^ TCID_50_s, and no mortality occurred at the low dose of 2 × 10^3^ TCID_50_s. However, all PBS-treated control mice survived without clinical symptoms during the entire observation period. In addition to lethality, the Abx-treated mice infected with 2 × 10^7^ TCID_50_s of EMCV developed various clinical symptoms, including hunched posture, trembling, hind limb paralysis, dyspnea, and death at 5 dpi, while the PBS-treated mice exhibited no obvious clinical signs (Fig. 1A).

We then harvested the blood and brain tissues of the infected mice at 3 and 5 dpi for virus load detection using real-time reverse transcription-PCR (RT-PCR). It was shown that peroral EMCV inoculation resulted in significantly higher viral burdens in the blood and brain tissues of Abx-treated mice than the PBS-treated mice, indicating that viral replication in the circulatory system and the target tissues was controlled by the presence of microbiota during acute EMCV infection via oral delivery (Fig. 1B). Consistent with the clinical symptoms and viral burden, immunofluorescent assays revealed that there were more viral signals (minor capsid protein VP2; Fig. 1C, left, magenta) in the brain sections of Abx-treated mice than the PBS-treated mice at 5 dpi (Fig. 1C, right). It seems that alteration of microbiota composition does not alter the cellular tropism of EMCV in the brain, because the majority of viral signals were still costained with the marker of astrocytes (glial fibrillary acidic protein [GFAP]; Fig. 1C, left, red) but not with the marker of microglia (IBA-1; green) in either PBS- or Abx-treated mice. Collectively, these data indicate that the intestinal microbes are essential for limiting EMCV replication in the target cells in the brain and are required to protect against neurological disease and cerebral tissue lesions from EMCV peroral infection.

To further confirm that the intestinal microbiome had an extraintestinal effect on limiting EMCV systemic infection, we next infected both PBS- and Abx-treated mice systemically with 200 TCID_50_s of EMCV in 100 μl inoculum via intraperitoneal injection to analyze the viral neurovirulence through this infectious route. Indeed, Abx-treated mice were more susceptible to intraperitoneal infection than PBS-treated animals, and reintroduction of fecal bacteria into Abx-treated animals alleviated EMCV mortality and disease (Fig. 1D). As the onset of viral dissemination and *in vivo* replication is faster in the context of intraperitoneal inoculation than peroral infection, we measured the viral loads in the blood and brain tissues earlier at 1 and 3 dpi and discovered that the Abx-treated mice supported more significant EMCV replication than PBS-treated mice, whereas viral titers of Abx-treated mice that had undergone fecal microbiota transplantation (FMT) were similar to those of PBS-treated mice (Fig. 1E). It was demonstrated that EMCV viremia and replication in target tissues were greatly increased in the absence of microbiota and that FMT could restore the capability for systemic viral clearance. Taken together, these data indicate that commensal bacteria are critical in promoting host systemic immunity against viral infection beyond the intestinal mucosal barrier.

### Microbiota deficiency severely diminishes innate mononuclear phagocytes’ antiviral immune responses to systemic EMCV infection.

**FIG 2:**
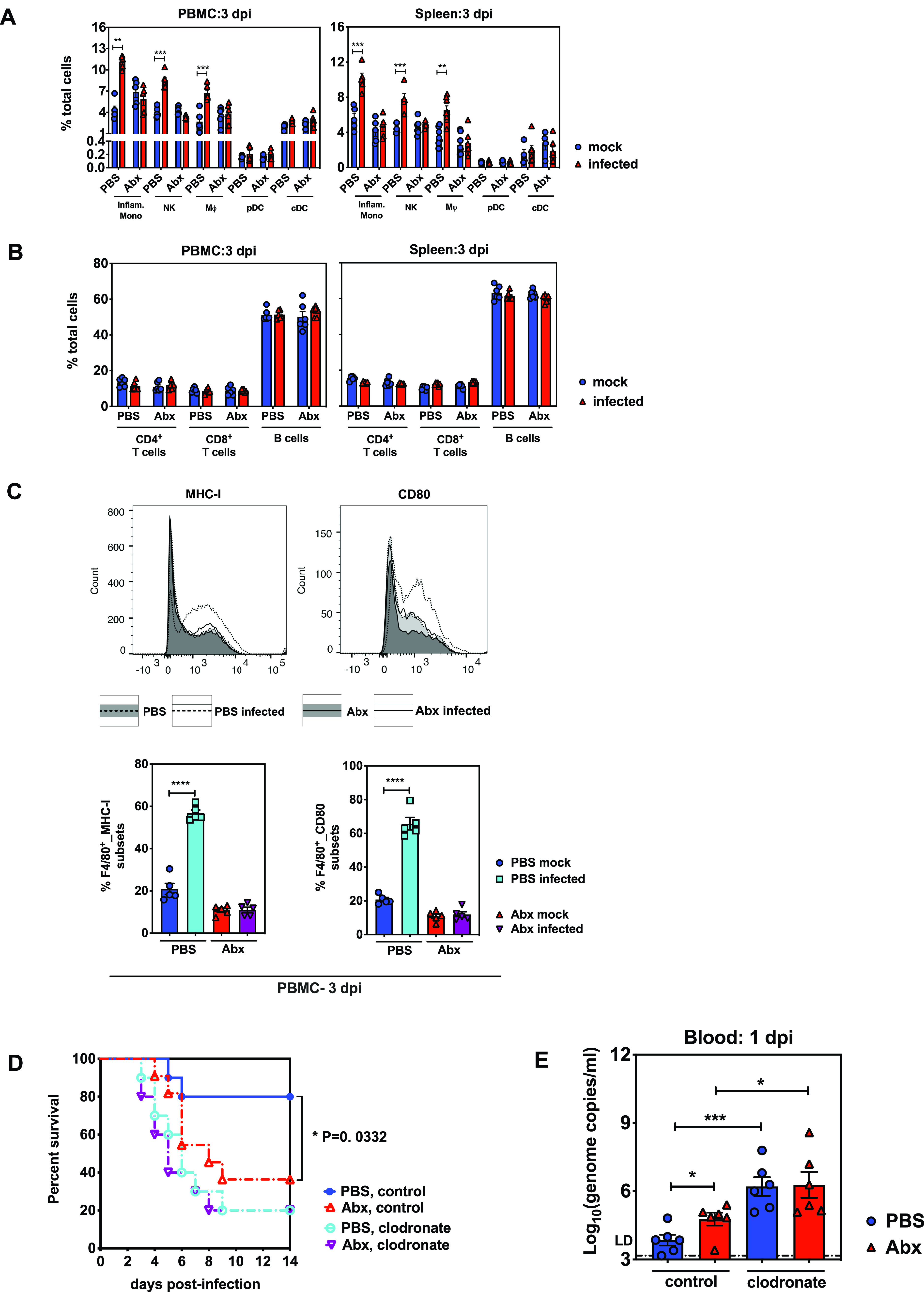
Innate cellular immune responses were severely diminished in Abx-treated mice after EMCV infection. (A) Frequency of a panel of innate immune cells, including inflammatory monocytes, NK cells, mononuclear phagocytes, pDCs, and cDCs, in PBMCs or splenocytes of mock-infected or infected PBS- or Abx-treated mice at 3 dpi (*n* = 4 to 8). (B) Frequency of CD4^+^ T cells, CD8^+^ T cells, or B cells from PBMCs or spleens of mock-infected or infected PBS- or Abx-treated mice at 3 dpi (*n* = 6). (C) Expression of MHC-I and CD80 on PBMC mononuclear phagocytes from mock-infected or infected PBS- or Abx-treated mice at 3 dpi (top two panels, histograms of MHC-I- or CD80-expressing mononuclear phagocytes; bottom two panels, frequency of mononuclear phagocytes expressing MHC-I or CD80 in total PBMC mononuclear phagocytes; *n* = 6). (D) Survival analysis of PBS- or Abx-treated mice treated with clodronate liposomes or control reagent following EMCV intraperitoneal inoculation (*n* = 10 or 11). (E) Blood viral titers of PBS- or Abx-treated mice treated with clodronate liposomes or control reagent at 1 dpi (*n* = 6). Data are from two independent experiments, and *P* values were determined by unpaired two-tailed Student's *t* test. *, *P* < 0.05; **, *P* < 0.01; ***, *P* < 0.001; ****, *P* < 0.0001.

The impaired viral clearance in the blood and brains of Abx-treated animals after EMCV infection led to the hypothesis that the early systemic innate immune response was crippled by microbiota depletion. Thus, we first evaluated the recruitment and activation of early responding innate immune cells after EMCV infection by infecting PBS-or Abx-treated mice intraperitoneally with 200 TCID_50_s of EMCV and extracting the peritoneal blood mononuclear cells (PBMCs) and splenocytes at 3 dpi. The cell extracts were stained with different antibodies specific for various cell types and quantified by flow cytometry. Compared with the respective mock-infected control mice, infected PBS-treated mice exhibited a significant increase in the percentages of inflammatory monocytes, NK cells, and mononuclear phagocytes, whereas there was a comparable frequency of pDCs and cDCs. In contrast with these data, it seems that EMCV infection did not activate any of these cell subsets in infected versus mock-infected Abx-treated mice (Fig. 2A). Also, analysis of the T cells and B cells suggests that depletion of commensal bacteria did not alter the frequencies of T/B lymphocytes in the context of EMCV infection (Fig. 2B).

Because of its crucial role as a cytotoxic effector molecule produced by NK cells, we next tested whether the production of granzyme B (GrB) was hindered in the microbiota-deficient mice. Respective gating strategies are presented in Fig. S1A in the supplemental material. As shown in Fig. S1B, NK cells from Abx-treated infected mice did not excrete upregulated GrB at 3 dpi compared to Abx-treated mock-infected mice, while EMCV infection induced remarkable excretion of GrB in PBS-treated mice. These data indicated that the cytotoxicity of NK cells was greatly impaired without commensal bacteria during viral systemic infection.

10.1128/mBio.00366-21.2 FIG S1 Flow cytometry gating strategy of NK cells in PBMC of mock-infected or infected mice with and without Abx treatment at 3 dpi (related to Fig. 2). (A) Representative flow cytometry plots showing the gating scheme for NK1.1-PE-positive cells in PBMC isolated from PBS-treated mock-infected, Abx-treated mock-infected, PBS-treated infected, and Abx-treated infected mice. (B) Frequency and expression of granzyme B (GrB) on PBMC NK cells from PBS- and Abx-treated mice at 3 dpi. Download FIG S1, PDF file, 1.3 MB. Copyright © 2021 Yang et al. 2021 Yang et al. https://creativecommons.org/licenses/by/4.0/ This content is distributed under the terms of the Creative Commons Attribution 4.0 International license.

Previous studies have shown that NK cell priming is dependent on cytokines expressed by IFN-I-stimulated mononuclear phagocytes, including DCs and macrophages (13, 21). Macrophages, but not DCs, had decreased expression of surface molecules that are critical during the early responses to lymphocytic choriomeningitis virus (LCMV) or influenza virus infection under the condition of Abx oral treatment (15). Consistent with these findings, we discovered that surface molecules reflecting macrophage activation, like major histocompatibility complex class I (MHC-I) and CD80, were greatly augmented in the PBMCs from infected PBS-treated mice but not in those from infected Abx-treated mice at 3 dpi compared to mock-infected animals (Fig. 2C and Fig. S2). To further determine the requirement for mononuclear phagocytes in the systemic antiviral innate immunity and verify the necessity of the intestinal microbiome in the mononuclear phagocytes’ response to viral infection, we treated mice given PBS or Abx systemically with clodronate liposomes to deplete mononuclear phagocytes and analyzed survival kinetics after intraperitoneal EMCV inoculation. Depletion of mononuclear phagocytes was at least 90% effective, as assessed by flow cytometry (Fig. S3). For the mice that received control reagent, Abx treatment still resulted in significantly higher death rates than PBS controls; however, for the mice injected with clodronate liposomes, Abx treatment did not make the animals more susceptible to EMCV infection (Fig. 2D). In support of this observation, at 1 dpi, higher titers of EMCV were detected in the blood of PBS-treated mice than Abx-treated mice both given the control reagent, and no difference was observed between PBS-treated and Abx-treated mice given clodronate liposomes (Fig. 2E). Colonized mice treated with clodronate liposomes had almost 100-fold-higher blood viral loads than control reagent-treated colonized mice, indicating that mononuclear phagocytes are absolutely important for the early control of EMCV systemic infection. Additionally, blood viral titers in mice depleted of mononuclear phagocytes and commensal bacteria were also significantly higher than those in Abx-treated mice without mononuclear phagocyte depletion, suggesting that commensal bacteria are less important than mononuclear phagocytes in conferring protection against EMCV infection (Fig. 2E).

10.1128/mBio.00366-21.3 FIG S2 Flow cytometry gating strategy of mononuclear phagocytes in PBMC of mock or infected mice with and without Abx treatment at 3 dpi (related to Fig. 2). Representative flow cytometry plots showing the gating scheme for F4/80-CD11b double-positive cell subsets in PBMCs isolated from PBS-treated mock-infected mice and representative histogram image of MHC-I^hi^ (A) or CD80 (B) staining on the surface of these cell subsets (bottom left); the same gating strategy was utilized for all mouse groups, and overlaid histogram images are presented (bottom right). Download FIG S2, PDF file, 0.7 MB. Copyright © 2021 Yang et al. 2021 Yang et al. https://creativecommons.org/licenses/by/4.0/ This content is distributed under the terms of the Creative Commons Attribution 4.0 International license.

10.1128/mBio.00366-21.4 FIG S3 Depletion of splenic mononuclear phagocytes following administration of clodronate liposomes (related to Fig. 2). Histograms showing CD11b^+^ cells isolated from spleen of mice treated with control reagents (A) or clodronate liposomes (B). Download FIG S3, PDF file, 0.1 MB. Copyright © 2021 Yang et al. 2021 Yang et al. https://creativecommons.org/licenses/by/4.0/ This content is distributed under the terms of the Creative Commons Attribution 4.0 International license.

Together, these data suggest that gut microbiota might be correlated with the activation of mononuclear phagocytes, while disrupting the bacterial composition by antibiotic administration might result in a negative effect on the mononuclear phagocyte-mediated protective innate immunity that limits viral systemic infection.

### Commensal bacteria depletion greatly impairs the systemic and cerebral IFN-I response following EMCV infection.

**FIG 3:**
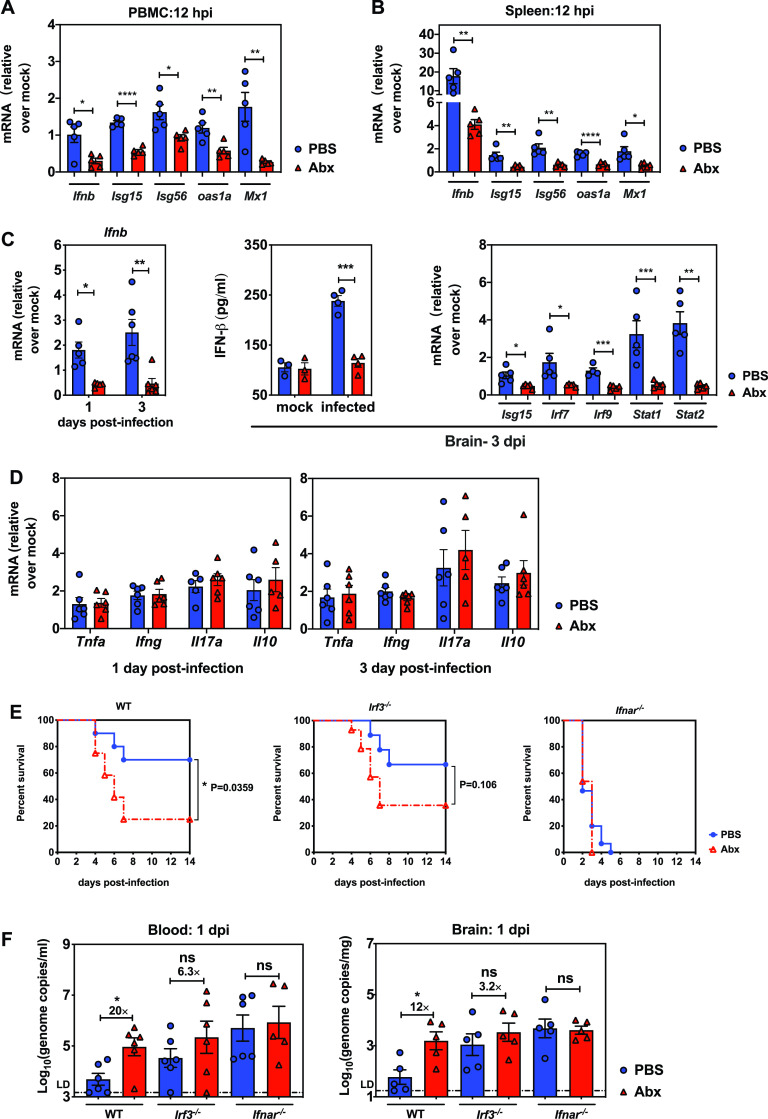
Microbiota depletion results in significantly impaired systemic or cerebral type I IFN responses. Fold induction of *Ifnb* and ISG expression in PBMC (A) and spleen (B) of PBS- or Abx-treated mice at 12 hpi following EMCV infection relative to respective mock controls (*n* = 5). (C) Expression of *Ifnb* and ISGs in the brains of infected PBS- or Abx-treated mice (fold change compared to respective mock-infected mice) at 1 or 3 dpi (*n* = 5 or 6). IFN-β levels in the brain at 3 dpi as detected by ELISA (middle). (D) Fold induction of proinflammatory or immune regulatory cytokine expression in the brain at 1 or 3 dpi relative to respective mock-infected mice (*n* = 6). (E) Survival kinetics of PBS- or Abx-treated *Irf3*^−/−^ or *Ifnar*^−/−^ mice infected with EMCV intraperitoneally; wild-type C57BL/6J mice served as controls (left, *n* = 10 to 12; middle, *n* = 9 to 14; right, *n* = 13 to 15). (F) Viral titers in blood and brain collected from infected PBS- or Abx-treated WT, *Irf3*^−/−^, or *Ifnar*^−/−^ mice at 1 dpi (*n* = 5 or 6). All data are from two independent experiments. *, *P* < 0.05; **, *P* < 0.01; ***, *P* < 0.001; ****, *P* < 0.0001; ns, not significant.

Because IFN-I is the key factor that contributes to the stimulation of macrophages, which subsequently prime NK cells for antigen encounter via cytokine expression, we hypothesized that the microbiota could regulate the IFN-I response in our model. To test whether the commensal bacteria altered the profile of IFN-I production at different time points postinfection, we inoculated groups of Abx-treated WT mice and PBS-treated controls with 200 TCID_50_s of EMCV intraperitoneally, collected blood and spleen at various time points postinfection, and probed for IFN-β and ISGs by real-time PCR. As early as 12 h postinfection (hpi), there was significantly upregulated expression of IFN-β and ISGs, including *Isg15*, *Isg56*, *Oas1a*, and *Mx1*, in the PBMC extract (Fig. 3A) and spleens (Fig. 3B) of PBS-treated mice compared to Abx-treated mice, suggestive of an impaired innate immune response mediated by IFN-I. Consistent with the affected systemic IFN-I response postinfection, there was reduced expression of IFN-β and associated antiviral defense genes like *Isg15*, *Irf7*, *Irf9*, and *Stat1/2* in the brains of Abx- versus PBS-treated mice at 1 and 3 dpi, indicating that the antiviral innate immunity is impeded not only systemically but also locally at the target site of dissemination (Fig. 3C). In contrast, no differences in expression of proinflammatory cytokine tumor necrosis factor alpha (TNF-α), type II interferon, and the immune regulatory cytokines interleukin 17 (IL-17) and IL-10 were observed in the brain tissues of PBS- and Abx-treated mice at 3 dpi, implying that the impaired antiviral immunity in microbiome-deficient Abx-treated mice was not associated with the production of these cytokines (Fig. 3D).

To further confirm that the commensal-bacterium-driven type I interferon response is critical in antiviral innate immunity, we examined the requirement for the IFN-I induction and amplification pathway in early EMCV control with or without antibiotic suppression. *Irf3*^−/−^ or *Ifnar*^−/−^ mice, which lack key mediators in either the type I interferon upstream induction pathway or the amplification loop, respectively, were utilized to clarify the effects of microbiota on different phases of IFN-I production during EMCV systemic infection. Despite a narrower difference between PBS- and Abx-treated wild-type mice (*P* = 0.0359), the lethality rate discrepancy in *Irf3*^−/−^ mice was still above 30% (*P* = 0.106). In contrast, all *Ifnar*^−/−^ mice succumbed to EMCV inoculation by 5 dpi, showing identical susceptibility to EMCV intraperitoneal infection regardless of PBS or antibiotic treatment (Fig. 3E). Consistent with the survival kinetics, the dissimilarities between PBS and Abx wild-type mice in viremia (20-fold) and brain viral replication (10-fold) at 1 dpi narrowed to 6.3-fold and 3-fold in *Irf3*^−/−^ mice, whereas the differences were completely lost in *Ifnar*^−/−^ mice (Fig. 3G). These findings indicate that the *Irf3*-mediated IFN-I induction pathway was partially affected by Abx treatment. Given the fact that *Ifnar*^−/−^ mice are extremely susceptible to EMCV infection even at a low dose of 50 TCID_50_s with or without having an intestinal microbiome, we would assume that the IFN-I amplification pathway is absolutely essential for preventing EMCV systemic infection and the IFNAR-mediated pathway might be correlated with the integrity of the host intestinal microbiome.

### *B. coccoides* monocolonization alleviates EMCV pathogenesis and restricts viral infection.

**FIG 4:**
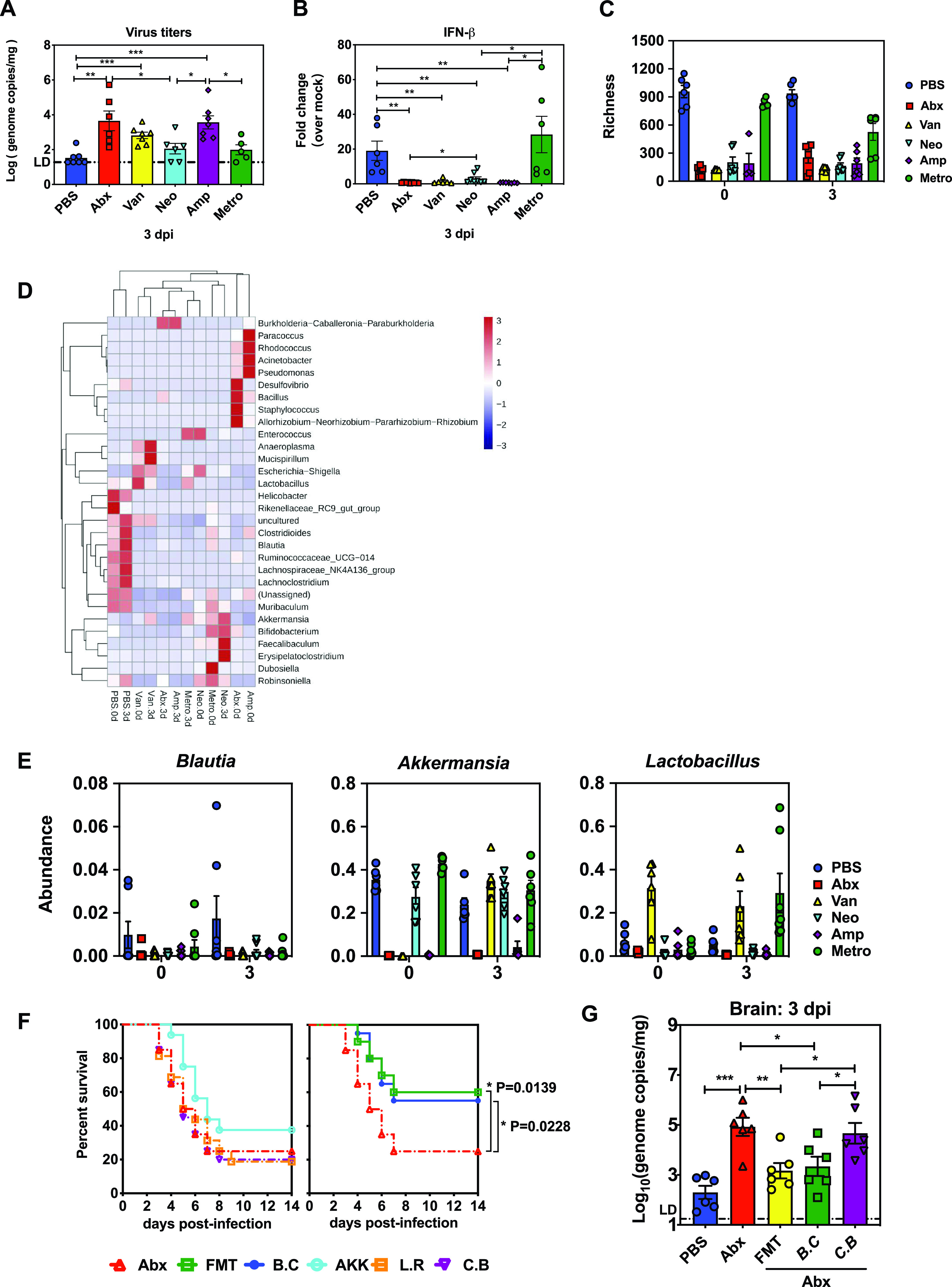
*B. coccoides* protects EMCV systemic infection by restricting viral *in vivo* replication. Viral titers (A) and induction of *Ifnb* (B) in the brains of PBS-, Abx-, or single antibiotic-treated mice were measured by qPCR at 3 dpi (*n* = 5 or 6). (C) Bacterial richness was defined by the number of unique taxa. (D) Heat map of relative abundance from different antibiotic treatment groups. (E) Relative abundance changes of bacteria at the genus level. (F) Survival analysis of Abx-treated mice colonized with *B. coccoides* (B.C), *A. muciniphila* (AKK), L. reuteri (L.R), *C. butyricum* (C.B), or mouse fecal contents (FMT) following EMCV intraperitoneal inoculation (3 experiments; *n* = 16 to 20). (G) Viral burden in brain tissues harvested from mock-infected or infected mice that were PBS treated, Abx treated, or Abx treated and colonized with FMT, *B. coccoides*, or *C. butyricum* at 3 dpi (*n* = 6). All data are from at least two independent experiments. *, *P* < 0.05; **, *P* < 0.01; ***, *P* < 0.001.

We next investigated whether specific commensal bacteria taxa are responsible for the protein against systemic EMCV infection. First, we tested this idea by determining which antibiotic in the cocktail was required for the effect on IFN-I modulation and whether the effect could be narrowed down to a single antibiotic treatment or to a certain kind of alteration in the intestinal microbiome *per se*. Groups of WT B6 mice were treated with a combination of four antibiotics or with Van, Neo, Amp, or Metro singly and inoculated intraperitoneally with EMCV, and the brain tissues were dissected at 3 dpi for EMCV viral burden measurement and IFN-β expression detection. Generally, single-Abx treatment significantly increased viral replication in the brain compared to PBS-treated controls except Metro or Neo. Van treatment exhibited a partial suppressive effect in early EMCV control, as brain viral titers of these mice were not as high as those in Abx cocktail-treated animals. Amp was the most effective single antibiotic, as the viral replication was comparable to that seen with the Abx combination (Fig. 4A).

Consistent with these data, Metro-treated mice exhibited levels of IFN-β expression very similar to those of the Abx cocktail-treated controls, suggesting that Metro was not required for the IFN-β suppressive effect. Amp, Van, and Neo treatments all dampened IFN-β expression significantly compared to PBS. Among them, Amp treatment conferred an optimal suppression on IFN-β expression level, Van treatment showed an intermediate effect, while Neo treatment had the least impact on IFN-β expression (Fig. 4B). As expected, successful inhibition of IFN-β expression in brain tissues in general correlated with a substantial reduction in detectable 16S rRNA readouts upon antibiotic treatment (Fig. 4C). In comparison, Van, Neo, or Amp treatment had an effect similar to that of the antibiotic cocktail, whereas Metro exhibited the least effective bacterial depletion (Fig. 4C). Based on these data, one would hypothesize that certain bacterial communities which were present in the PBS control and resistant to Metro treatment but significantly lower in the Neo-, Van-, Amp-, and Abx-treated mice mediated the protective effect against EMCV systemic infection as an IFN-I inducer. Thus, we then carried out 16S rRNA gene sequencing and analysis on fecal samples collected from PBS-, Abx cocktail-, and single-antibiotic-treated mice at 0 and 3 dpi (Fig. S4 and Fig. 4D).

10.1128/mBio.00366-21.5 FIG S4 Heat map of bacterial relative abundance from different groups receiving PBS, Abx cocktail, and four single-antibiotic treatments prior to EMCV infection (referred to as 0 dpi; related to Fig. 4). Download FIG S4, PDF file, 1.1 MB. Copyright © 2021 Yang et al. 2021 Yang et al. https://creativecommons.org/licenses/by/4.0/ This content is distributed under the terms of the Creative Commons Attribution 4.0 International license.

On the basis of the hypothesis mentioned above, we chose three bacterial taxa, namely, *Blautia*, *Akkermansia*, and *Lactobacillus*, because they had similar abundances in PBS- and Metro-treated mice at 0 and 3 dpi in general (Fig. 4E). To explore whether they are the specific bacteria that mediated the protective effect of the microbiota against viral systemic infection, we subjected Abx-treated mice to gavage with *B. coccoides*, Akkermansia muciniphila, Lactobacillus reuteri (an unrelated Gram-positive human symbiont), Clostridium butyricum, or mouse fecal contents (fecal microbiota transplantation [FMT]) prior to EMCV intraperitoneal inoculation. Strikingly, colonization of Abx-treated mice with *B. coccoides* alone fully alleviated EMCV mortality to the extent seen in mice that underwent FMT and that of PBS-treated controls (Fig. 4F), whereas gavage with *C. butyricum*, L. reuteri, or A. muciniphila did not alter mortality, even though these bacteria exhibited efficient colonization (Fig. S4). Similarly, we observed significantly lower viral burdens in the brain tissues of *B. coccoides*-colonized Abx-treated mice than in Abx-treated, FMT recipient, or *C. butyricum*-colonized Abx-treated mice at 3 dpi (Fig. 4G). Taken together, these data suggest that the specific commensal *B. coccoides* protects hosts from systemic EMCV infection by restricting viral replication.

### *B. coccoides* monocolonization promotes macrophage activation and IFN-I responses to systemic EMCV infection.

To further elucidate whether *B. coccoides* restricted EMCV infection by promoting macrophage activation, we then subjected Abx-treated mice to gavage with *B. coccoides*, *C. butyricum*, or fecal contents to evaluate GrB-secreting NK cells and mononuclear phagocytes with activation of the surface markers MHC-I and CD80 in PBMC and spleen. Indeed, *B. coccoides* colonization in Abx-treated mice restored frequency of GrB-secreting NK cells in PBMCs following EMCV infection to the level of PBS-treated controls at 3 dpi, whereas *C. butyricum* oral gavage did not improve the impairment of GrB secretion in NK cells exhibited in Abx-treated controls during infection (Fig. S5).

10.1128/mBio.00366-21.6 FIG S5 *B. coccoides* colonization restores the cytotoxicity of NK cells. Frequency of NK cells expressing granzyme B (GrB) in PBMCs or spleen isolated from PBS-treated mice, Abx-treated mice, or Abx-treated mice colonized with FMT, *B. coccoides*, or *C. butyricum* at 3 dpi following EMCV inoculation (*n* = 6). Download FIG S5, PDF file, 0.04 MB. Copyright © 2021 Yang et al. 2021 Yang et al. https://creativecommons.org/licenses/by/4.0/ This content is distributed under the terms of the Creative Commons Attribution 4.0 International license.

**FIG 5:**
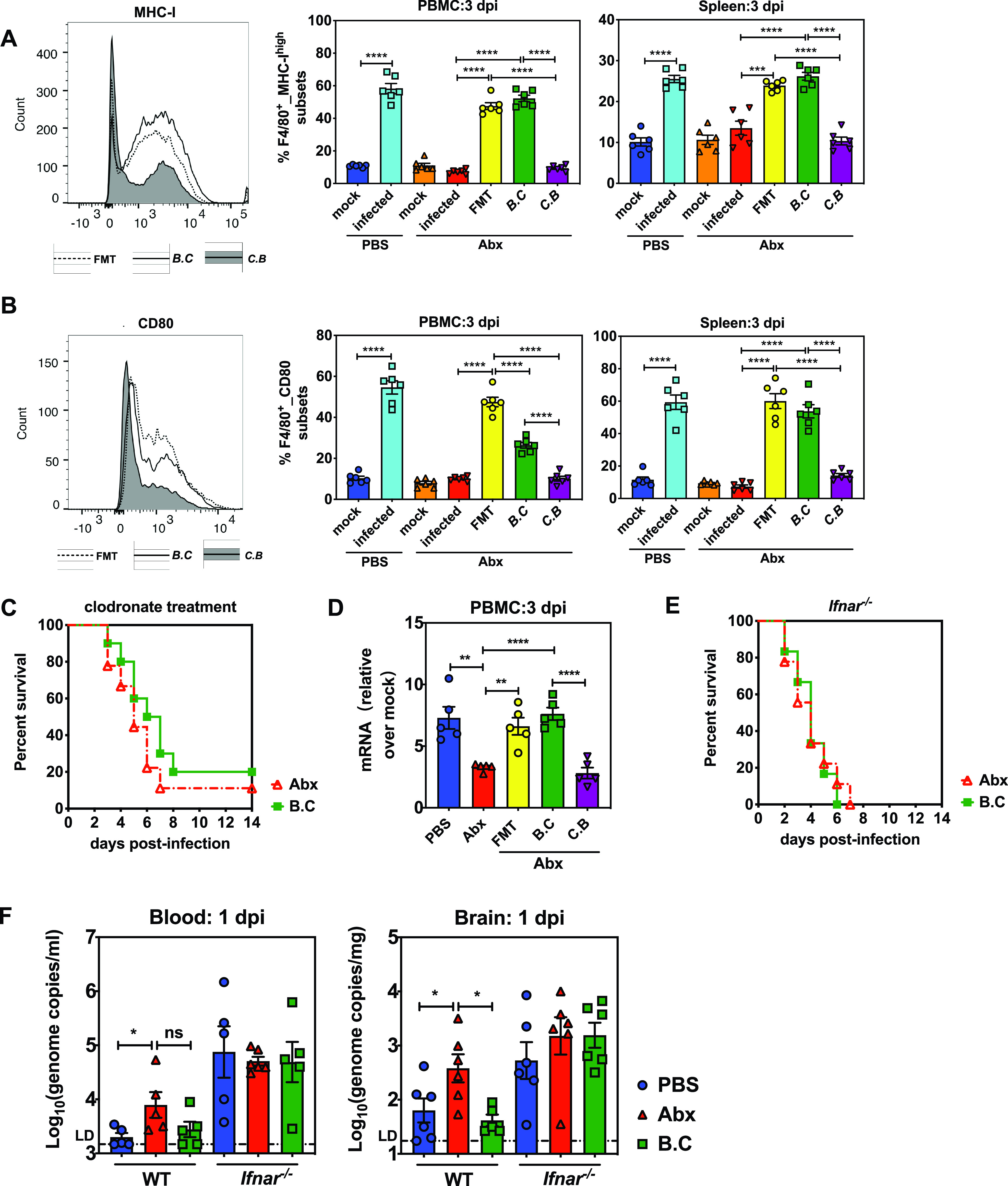
*B. coccoides* colonization restricts enteric virus systemic infection by activating innate cellular immune responses and type I IFN expression in a macrophage- and *Ifnar*-dependent manner. Frequency of mononuclear phagocytes expressing MHC-I (A) or CD80 (B) in PBMC or spleen isolated from PBS-treated mice, Abx-treated mice, or Abx-treated mice colonized with FMT, *B. coccoides*, or *C. butyricum* at 3 dpi following EMCV inoculation (representative flow cytometry histograms showing surface staining of MHC-I or CD80; *n* = 6). (C) Survival kinetics of infected Abx-treated and *B. coccoides*-colonized Abx-treated mice injected with clodronate liposomes (*n* = 9 or 10). (D) Expression level of *Ifnb* in PBMCs isolated from infected PBS-treated mice, Abx-treated mice, or Abx-treated mice subjected to gavage with FMT, *B. coccoides*, or *C. butyricum*, compared to their respective mock-infected controls (*n* = 5). (E) Survival kinetics of infected Abx-treated and *B. coccoides*-colonized Abx-treated *Ifnar*^−/−^mice (*n* = 6 to 9). (F) Viral titers in blood and brain collected from infected PBS-treated, Abx-treated, or *B. coccoides*-colonized WT or *Ifnar*^−/−^ mice at 1 dpi (*n* = 5 or 6). All data are from two independent experiments, and *P* values were determined by unpaired two-tailed Student's *t* test. *, *P* < 0.05; **, *P* < 0.01; ***, *P* < 0.001; ****, *P* < 0.0001; ns, not significant.

*B. coccoides* colonization in Abx-treated mice remarkably increased the frequency of MHC-I mononuclear phagocytes by 45% in PBMC (7.2% to 52%) and by 13% in spleen (from 13.5% to 26%) monocytes/macrophages following EMCV infection at 3 dpi, comparable to FMT recipient mice and PBS-treated controls (Fig. 5A). In a similar way, levels of CD80-upregulated mononuclear phagocytes were elevated from 10% to 26.4% in PBMCs and from 7.36% to 53.7% in spleen mononuclear phagocytes after *B. coccoides* colonization in the context of EMCV infection (Fig. 5B). *B. coccoides* colonization recovered MHC-I expression in both PBMC and spleen mononuclear phagocytes almost to the same extent as in FMT recipient mice and PBS-treated controls, whereas it only partially rescued CD80 expression, to a lesser extent than FMT, especially in PBMC mononuclear phagocytes (Fig. 4B) (16.4% versus 37.5%), indicating that the expression of this surface molecule might not be regulated only by *B. coccoides* but might also be dependent on other intestinal bacteria. Likewise, *C. butyricum*-colonized mice retained a low level of either MHC-I- or CD80-upregulated PBMC and spleen mononuclear phagocytes compared to Abx-treated mice following viral infection at 3 dpi. To determine whether *B. coccoides* mediates protection from systemic EMCV infection through a mononuclear phagocyte-dependent mechanism, we then colonized mononuclear phagocyte-depleted Abx-treated mice with *B. coccoides* before intraperitoneal EMCV inoculation and observed the survival curves for 14 days. Clodronate treatment abolished *B. coccoides*-mediated protection, demonstrating that *B. coccoides* is required for conferring mononuclear phagocyte-mediated protection against EMCV infection (Fig. 5C).

To determine whether colonizing Abx-treated mice with *B. coccoides* restores systemic IFN-I responses to viral infection, we harvested PBMCs from Abx-treated mice with *B. coccoides*, *C. butyricum*, or fecal content colonization at 3 dpi and measured IFN-β expression by qPCR. Apparently, *B. coccoides* colonization in Abx-treated mice restored IFN-β expression in PBMCs to the level detected in FMT recipient and PBS-treated controls upon EMCV infection at 3 dpi, whereas *C. butyricum* colonization did not (Fig. 5D). Since we have demonstrated that IFN-I amplification pathway is absolutely essential for antagonizing viral systemic infection, we next investigated whether *B. coccoides* mediates protection from EMCV infection in an IFN-I-dependent manner. We colonized *Ifnar*^−/−^ mice with *B. coccoides* prior to virus inoculation and discovered that these mice did not display less susceptibility to EMCV infection than Abx-treated *Ifnar*^−/−^ mice (Fig. 5E). Furthermore, *B. coccoides* colonization of Abx-treated *Ifnar*^−/−^ mice did not reduce viremia or brain replication in comparison to Abx-treated *Ifnar*^−/−^ controls at 1 dpi (Fig. 5F). As a whole, these data demonstrate that the protective effect of *B. coccoides* against viral systemic infection requires mononuclear phagocytes and IFN-I amplification signaling.

### *B. coccoides* colonization promotes type I IFN responses in mononuclear phagocytes to limit EMCV infection.

**FIG 6:**
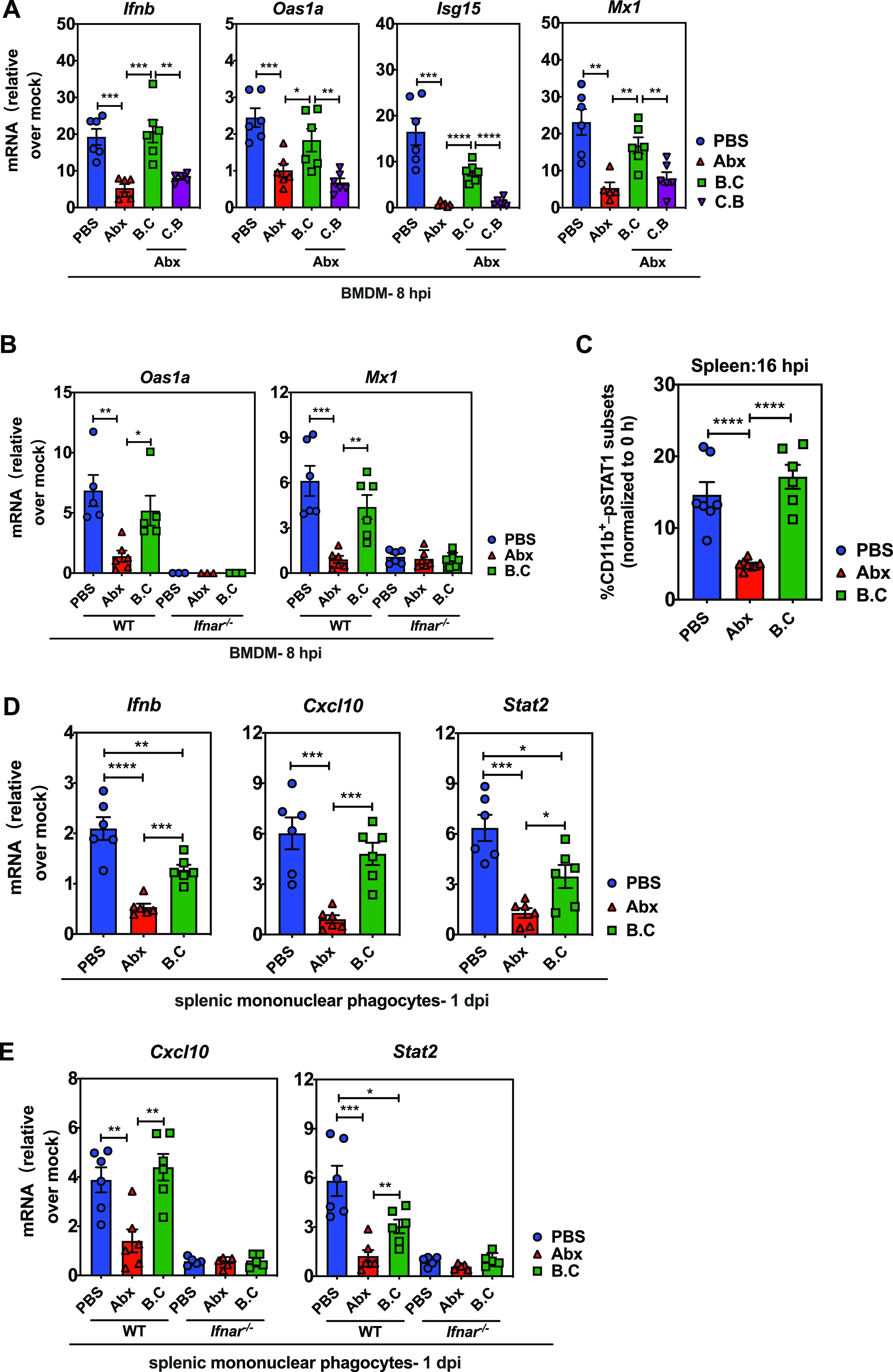
*B. coccoides* colonization promotes type I IFN and ISG responses in mononuclear phagocytes to limit EMCV infection. (A) *Ifnb* and antiviral defense gene expression in BMDMs isolated from PBS-treated, Abx-treated, or *B. coccoides*- or *C. butyricum*-colonized Abx-treated ice at 8 h after EMCV infection (MOI = 5) *in vitro* (*n* = 5 or 6). (B) Relative mRNA expression of *Oas1a* and *Mx1* in BMDMs isolated from WT or *Ifnar*^−/−^ mice that pretreated with PBS, Abx, or Abx plus *B. coccoides* colonization at 8 hpi *in vitro* (*n* = 5 or 6). (C) Splenic mononuclear phagocytes isolated from spleens of PBS-treated, Abx-treated, or *B. coccoides*-colonized Abx-treated mice were stimulated with 200 ng/ml IFN-γ *in vitro*. The data are the averaged frequencies of pSTAT1-stained CD11b^+^ cell subsets at 16 h minus the frequency of these cells tested at 0 h after IFN-γ stimulation (3 experiments; *n* = 6). (D) *Ifnb* and antiviral defense gene expression in splenic mononuclear phagocytes isolated from EMCV-infected PBS-treated, Abx-treated, or *B. coccoides*-colonized Abx-treated mice at 1 dpi (*n* = 6). (E) Relative mRNA expression of *Cxcl10* and *Stat2* in splenic mononuclear phagocytes isolated from EMCV-infected WT or *Ifnar*^−/−^ mice pretreated with PBS, Abx, or Abx plus *B. coccoides* colonization at 1 dpi (*n* = 5 or 6). Data are from at least two independent experiments. *, *P* < 0.05; **, *P* < 0.01; ***, *P* < 0.001; ****, *P* < 0.0001.

To directly clarify whether *B. coccoides* can promote systemic type I IFN responses through monocyte/macrophage activation, we infected bone marrow-derived macrophages (BMDMs) isolated from PBS-treated, Abx-treated, *B. coccoides*-colonized, or *C. butyricum*-colonized mice with EMCV at a multiplicity of infection (MOI) of 5 *in vitro* and detected induction of *Ifnb* and associated ISGs at 8 hpi using quantitative RT-PCR (qRT-PCR). Expression of *Ifnb* and ISGs, including *Oas1a*, *Isg15*, and *Mx1*, was drastically reduced in BMDMs isolated from Abx-treated mice compared to PBS mice, suggestive of an intrinsic inability to respond to viral infection, but this responsiveness was partially restored by *B. coccoides*, but not *C. butyricum*, colonization (Fig. 6A). Furthermore, *B. coccoides* colonization in Abx-treated mice endowed their BMDMs with the ability to induce ISG expression in an *Ifnar*-dependent manner, because ISG expression was undetectable (or extremely low) in BMDMs isolated from *B. coccoides*-colonized *Ifnar*^−/−^ mice following EMCV infection at 8 hpi (Fig. 6B).

Because STAT1 conveys signals downstream of IFN receptor engagement and its phosphorylation and nuclear translocation mediate transcription of ISGs in the IFN-I amplification loop, we stimulated mononuclear phagocytes isolated from spleens of PBS-treated, Abx-treated, or *B. coccoides*-colonized Abx-treated mice with IFN-γ *in vitro* and tested STAT1 phosphorylation using flow cytometry. IFN-γ-stimulated mononuclear phagocytes isolated from Abx-treated mice exhibited significantly low levels of pSTAT1 compared to those from PBS-treated mice, while *B. coccoides* colonization in Abx-treated mice restored STAT1 phosphorylation in splenic mononuclear phagocytes to levels comparable to those in PBS controls (Fig. 6C and Fig. S7). We also examined expression of multiple antiviral defense genes in mononuclear phagocytes isolated from the spleens of infected PBS-treated, Abx-treated, and *B. coccoides*-colonized animals to confirm whether the same phenotype exists *in vivo* after viral infection. At 1 dpi, splenic mononuclear phagocytes from Abx-treated mice exhibited significantly decreased antiviral gene expression compared to those from PBS-treated mice, but *B. coccoides* colonization in Abx-treated mice induced significantly higher levels of expression of *Ifnb* and ISGs, although they were not fully restored to the level of PBS controls (Fig. 6D). This is consistent with the data showing that *B. coccoides* colonization partially restored the frequency of CD80-expressing mononuclear phagocytes (Fig. 5C) in PBMCs, implying that other commensal bacteria species also possess the ability to induce type I IFN and ISG responses in circulating mononuclear phagocytes.

10.1128/mBio.00366-21.8 FIG S7 Flow cytometry gating strategy of sorted splenic mononuclear phagocytes from PBS-treated, Abx-treated, or *B. coccoides*-colonized Abx-treated mice stained for pSTAT1 after IFN-γ stimulation *in vitro* (related to Fig. 6). Representative flow cytometry plots showing the sorting scheme for F4/80 CD11b double-positive cell subsets of splenocytes isolated from PBS mice (top panels) and a representative histogram of intracellular staining of pSTAT1 in sorted mononuclear phagocytes at 0 h after IFN-γ stimulation (bottom left); the same gating strategy was utilized for pSTAT1 staining of sorted splenic mononuclear phagocytes from PBS-treated, Abx-treated, and *B. coccoides*-colonized Abx-treated mice, and overlaid histogram images of pSTAT1 staining at 16 h after IFN-γ stimulation are presented (bottom right). Download FIG S7, PDF file, 0.3 MB. Copyright © 2021 Yang et al. 2021 Yang et al. https://creativecommons.org/licenses/by/4.0/ This content is distributed under the terms of the Creative Commons Attribution 4.0 International license.

Again, *B. coccoides* exerts its effects through IFNAR-mediated IFN-I signaling, because no detectable or obviously upregulated antiviral gene expression was observed in mononuclear phagocytes isolated from spleens of *B. coccoides*-colonized Abx-treated *Ifnar*^−/−^ mice (Fig. 6E). Overall, these findings suggest that *B. coccoides* is sufficient for maintaining optimal mononuclear phagocyte responsiveness to stimuli (cytokine and viral infection), and it seems very likely that *B. coccoides* augments type I IFN signaling in mononuclear phagocytes via IFNAR and STAT1 phosphorylation to protect the host from virus systemic infection.

## DISCUSSION

In the present study, we describe a crucial role for the commensal microbiota in modulating antiviral innate immunity to systemic enteric virus infection. In particular, the intestinal microbiome protects against systemic viral infection by promoting type I IFN responses in peripheral innate immune cells, mainly mononuclear phagocytes, via an *Ifnar*-correlated signaling pathway. Furthermore, we have identified the mechanism by which an understudied commensal bacterium, *B. coccoides*, restricts enteric virus systemic infection by enhancing type I IFN responses in mononuclear phagocytes.

Over the past 10 years, it has been gradually acknowledged that the intestinal microbiome can shape host antiviral immunity. Accumulating literature has shown that animals given cocktails of broad-spectrum antibiotics and GF mice have impaired innate and adaptive immune responses following infection with various viral pathogens, including LCMV, influenza virus, murine cytomegalovirus (MCMV), flaviviruses, vesicular stomatitis virus (VSV), or Chikungunya virus (CHIKV) (13, 15, 16, 18, 22). However, until now, all studies on mammalian enteric viruses have reported that the intestinal microbiome promotes virus infection (6, 7, 10, 20, 23), with two exceptions. One study, conducted by Grau et al., reported that *C. scindens* primes type III interferon induction to suppress murine norovirus (MNV) infection in proximal small intestines by bile acid biotransformation (19). Another example of commensal bacterium-mediated anti-enteric virus protection was provided by Shi et al., who demonstrated that segmented filamentous bacteria (SFB) prevent and cure rotavirus infection by accelerating epithelial cell turnover (24). Unlike norovirus and rotavirus infection, which cause only self-limited diseases, some enteric viruses, enterovirus 71 and EMCV for instance, can result in systemic infection and lead to a diverse array of neurological diseases (25, 26).

To determine whether the intestinal microbiota could be protective indirectly by regulating host antiviral innate immunity during enteric virus infection, we infected wild-type mice with EMCV orally, to mimic natural infection, or intraperitoneally, to immediately establish systemic infection. To our surprise, microbiota depletion with antibiotics exacerbated the neurological disease and enhanced viral replication upon EMCV infection for both routes of inoculation (Fig. 1). This outcome resembles the results of previous studies on the nonenteric viral pathogens mentioned above but not the milestone study conducted by Kuss et al. using poliovirus and reovirus, in which intestinal microbiomes and LPS were reported to promote viral replication and systemic pathogenesis (6). These discrepancies suggest that microbiota acts very differently in interacting with even two closely related enteric viruses (EMCV and poliovirus are both picornaviruses and have very similar viral structures). It is highly likely that the innate immune responses were also severely diminished by antibiotic treatment in their model (we used the same antibiotic regimens), whereas poliovirus replication *in vivo* was barely established to reflect the defects on host innate immunity in the absence of microbiota. Conversely, EMCV *in vivo* replication and dissemination do not require the microbiota in our model (Fig. 1B and C), so that the neuropathogenesis was diminished in the context of antiviral innate immunity being dampened by microbiota depletion.

Collectively, multiple studies have demonstrated that commensal bacteria are responsible for shaping host antiviral immunity beyond mucosal sites. GF mice and Abx-treated mice exhibited blunted type I interferon responses, which are required for optimizing functions of nonmucosal immune cells, including NK cells, macrophages, and CD8^+^ T cells (13, 14, 16, 22). In our study, we showed that mononuclear phagocytes isolated from Abx-treated mice did not display a higher frequency or upregulated expression of activating surface markers like MHC-I and CD80 at 3 dpi (Fig. 2A and D). Additionally, mononuclear phagocyte depletion equalized the discrepancies in mortality and viral replication between Abx-treated mice and PBS controls, indicating that mononuclear phagocytes are essential for microbiota-mediated protection from EMCV systemic infection (Fig. 2E and F). Our data are consistent with previous studies demonstrating that the microbiome can regulate antiviral macrophage responses by inducing the expression of type I IFN and ISG genes after influenza virus infection (15, 16). Similar to these studies, we also showed that microbiota depletion results in severely diminished type I IFN and ISG responses in both peripheral tissues and brain (Fig. 3A to D), which causes unrestricted virus replication and exacerbates neuropathogenesis and mortality. The impaired IFN-I production and ISG expression in the brain tissues of Abx-treated mice suggests major defects in responsiveness of microglia to viral infection, because they are the main sources of IFN production in the brain. The results are consistent with one paper reporting that microglia of GF mice were unable to produce various cytokines and chemokines upon stimulation (27). Our model revealed that the microbiota-driven IFN and ISG responses are partially dependent on *Irf*3-mediated IFN-I induction and also probably rely on the *Ifnar*-mediated signaling pathway (Fig. 3E and F).

Although accumulated evidence shows that the microbiota can promote ISG responses in multiple cell types, the cellular sources of type I IFN were less well defined until very recently, when two papers demonstrated that the microbiota constitutively induces type I IFN production and a basal level of ISG expression in pDCs at systemic sites (17, 18). In our study, we examined only the frequency of pDCs in PBMC or spleen following EMCV infection at 3 dpi, and we observed no differences between PBS- and Abx-treated mice (Fig. 2A). Similarly, Abt et al. reported that at 3 dpi following influenza virus infection, there was a comparable influx of pDCs into bronchiole alveolar lavage and the pDCs exhibited similar activation profiles in PBS- and Abx-treated mice (15). These seemingly discordant observations could be explained by the timing of detection, because pDCs respond to virus nucleic acids with massive and rapid secretion of IFN-I (1 to 3 h poststimulation) independently of the IFNAR-based feedback signaling, which is always required for most cell types in IFN-I production (28). Also, profiling the IFN-I and ISG expression in pDCs could be very subtle, especially at steady state (0 dpi), and using different microbiota-deficient mouse models (GF versus Abx-treated mice) may result in distinct phenotypic differences (17, 18). Generally, it is plausible that signals of commensal bacteria drive a very swift first wave of IFN-I (especially IFN-β) production in poised pDCs to activate NK cells, macrophages, and overall innate immune responses to viral infection.

Until now, only a few publications had begun to identify specific commensal bacterial species that play an important role in inducing type I IFN-mediated innate antiviral immunity (16, 18, 29, 30). Although the study by Abt and colleagues revealed an interplay between commensal bacteria and poised low-level tonic antiviral interferon signaling, they did not identify the specific bacterial species that regulates the “steady-state” readiness of antiviral pathways in macrophages (15). In order to identify the specific commensal bacteria that are responsible for the protein against EMCV systemic infection, we performed 16S rRNA gene sequencing and analysis on fecal samples collected from PBS-, Abx cocktail-, and single-antibiotic-treated mice. Interestingly, we noted that EMCV infection has a dramatic effect on the prevalence of certain microbes, including *Blautia*, *Clostridioides*, *Lachnoclostridium*, and others, even in the PBS control (Fig. 4D and Fig. S4). This result is distinct from the data reported by Nelson et al., who showed that the infection with the enteric virus MNV does not cause major disruptions in the murine intestinal microbiota (31). We speculated that this might be attributed to the peripheral IFN-I production induced by EMCV systemic infection, because Deriu et al. reported that influenza virus-induced type I IFNs produced in the lungs promoted the depletion of obligate anaerobic bacteria and the enrichment of *Proteobacteria* in the gut, leading to a secondary Salmonella infection (32).

Our work builds on the limited existing literature by identifying a less well documented commensal bacterium, *B. coccoides*, that plays a crucial role in protecting against enteric virus systemic infection through IFN-I induction. Abx-treated mice subjected to gavage with 10^8^ CFU of *B. coccoides* reached a relative bacterial abundance similar to that in the PBS-treated control, suggesting that this dose results in an effective colonization close to a physiological level (Fig. S6). Above that, colonization with 10^10^ CFU of *B. coccoides* appeared to reach a level almost 40-fold higher than that in the PBS control (Fig. S6), and colonialization almost fully restored the upregulation of MHC-I and CD80 on the surfaces of PBMC and spleen mononuclear phagocytes in Abx-treated animals following EMCV infection (Fig. 5B and C). Single colonization of *B. coccoides* in Abx-treated mice can also rescue the intrinsic defects in expression of IFN-I and key ISGs in BMDMs in an *Ifnar*-dependent manner following EMCV infection *in vitro* (Fig. 6A and B) or restore the STAT1 phosphorylation in splenic mononuclear phagocytes following IFN-γ stimulation *in vitro* (Fig. 6C). The present study adds to the previous work by demonstrating that colonization of Abx-treated mice with a single bacterium, *B. coccoides*, can restore the ability of mononuclear phagocytes to respond to type II IFN or virus stimulation.

10.1128/mBio.00366-21.7 FIG S6 Relative abundance of bacteria in fecal samples collected from single-bacterium-colonized mice. Groups of Abx-treated mice were colonized with different doses of *B. coccoides* (A) or 10^10^ CFU of *C. butyricum* (B), and fecal relative bacterial abundance at 48 h postcolonization was determined by 16S rRNA sequencing (*n* = 4 or 5). Download FIG S6, PDF file, 0.04 MB. Copyright © 2021 Yang et al. 2021 Yang et al. https://creativecommons.org/licenses/by/4.0/ This content is distributed under the terms of the Creative Commons Attribution 4.0 International license.

Since *B. coccoides* monocolonization in Abx-treated mice restored protection of EMCV systemic infection by restricting *in vivo* viral replication in a type I interferon-dependent manner (Fig. 4F and G and 5D and F), a next question will be, what does *B. coccoides* utilize to induce IFN-I responses in mononuclear phagocytes to confer antiviral systemic protection? Two recent reports suggest that *C. scindens-*derived metabolite deoxycholic acid or glycolipids of Bacteroides fragilis can impact host antiviral innate immunity by inducing type I IFN production and ISG expression in pDCs or a subset of colonic cDCs (18, 30). Also, Gutierrez-Merino et al. have reported that beneficial commensal lactic acid bacteria can trigger type I interferon production via direct recognition by the intracellular sensors of STING and MAVS (33).

Short-chain fatty acids (SCFA), primarily produced by the Gram-positive bacteria *Firmicutes* and *Bacteroidetes* through fermentation of undigested polysaccharides, are not only important local energy sources for gut microbiota and intestinal epithelial cells but also crucial regulators for shaping immune systems at extraintestinal sites (5). As the most abundant SCFA in the colon, acetogenic bacterial communities such as *Blautia* produce nearly one-third of the acetate (34). It was shown that acetate derived from high-fiber diets protects against respiratory syncytial virus infection by promoting type I IFN production and ISG expression in pulmonary epithelial cells through GPR43 (35). Future studies will be carried out to examine the effects of specific SCFAs on host innate immunity to enteric viruses using a microbiota-deficient mouse model.

## MATERIALS AND METHODS

### Cell, bacterial isolates, and virus.

BHK-21 cells were cultured in Dulbecco’s modified Eagle medium (DMEM) supplemented with 10% fetal bovine serum (GIBCO, Invitrogen Corporation, Carlsbad, CA, USA) at 37°C and 5% CO_2_.

*B. coccoides* was purchased from ATCC (ATCC 29236) and cultured in modified chopped-meat medium (ATCC medium 1490; ELITE-MEDIA) at 37°C under anaerobic conditions. *C. butyricum* was purchased from ATCC (ATCC 19398) and cultured in thioglycolate medium (Hopebio) at 37°C under anaerobic conditions. *A. muciniphila* was kindly provided by Lan-juan Li (The First Affiliated Hospital, College of Medicine, Zhejiang University, Hangzhou, People’s Republic of China) and cultured in brain heart infusion medium (BHI; Oxoid) at 37°C under anaerobic conditions. L. reuteri was purchased from China Center for Type Culture Collection (CCTCC; AB 2014289) and cultured in DeMan-Rogosa-Sharpe medium (Oxoid) at 37°C under anaerobic conditions. The concentration of each bacterial species was quantified based on the optical density at 600 nm (OD_600_).

Recombinant virus derived from full-length clone of EMCV strain BJC3 (GenBank accession no. DQ464062) was used in this study (36), and viral titers of EMCV stocks were determined by a standard TCID_50_ assay described previously (37).

### Mice and infections.

Mice were maintained in a specific-pathogen-free (SPF) facility with a temperature- and humidity-controlled environment (22 ± 2°C, 50% ± 10% humidity), and all animal experiments were strictly carried out in accordance with protocols approved (no. 117113) by the Institutional Animal Care and Use Committee of Zhejiang University. Six- to 8-week-old, sex-matched mice were used for all experiments. C57BL/6J wild-type mice were purchased from the Model Animal Research Center of Nanjing University (Nanjing, China). Type 1 interferon receptor knockout mice (referred to as *Ifnar*^−/−^) were a kind gift from Yu Chen (Wuhan University, Hubei, People’s Republic of China), and interferon regulatory factor 3-deficient mice (referred to as *Irf3*^−/−^) mice were provided by Jin Jin (Zhejiang University, People’s Republic of China).

For all studies with wild-type B6 mice, animals were inoculated orally with 2 × 10^7^, 2 × 10^5^, or 2 × 10^3^ TCID_50_s of EMCV in 25 μl inoculum or infected intraperitoneally with 200 TCID_50_s of EMCV in 200 μl inoculum. For EMCV virulence assays on *Ifnar*^−/−^ mice, *Irf3*^−/−^ mice, or wild-type mice treated with clodronate liposomes, animals were inoculated intraperitoneally with 50 TCID_50_s of EMCV in 200 μl inoculum. Clinical symptoms were observed and documented blindly using the following scoring criteria: 0, normal; 1, hunched posture and trembling; 2, hind limb paralysis; 3, dyspnea and lack of responsiveness to touch; 4, sudden death.

For viral titer determination, tissue samples taken at different time points were harvested, weighed, and homogenized with stainless steel beads in 1 ml of DMEM supplemented with 2% fetal bovine serum (FBS) and titrated by qPCR. Briefly, tissue samples were homogenized at 45 Hz for 1 min, and the homogenates were clarified by centrifugation at 12,000 rpm for 5 min. Total RNA was extracted with TRIzol reagent (Invitrogen) and subjected to quantitative reverse transcriptase PCR (qRT-PCR) using one-step qPCR kits (Toyobo) on an ABI 7500 Fast instrument. Standard cycling conditions and primers and probe (forward primer, 5′-TGAGTCATTAGCCATTTCAACCCA-3′; reverse primer, 5′-CGTGAGATACAAACCCGCCCTA-3′; probe, 5′-TCCCATCAGGTTGTGCAGCGA-3′) were described previously (38). Viral burden was expressed on a log_10_ scale as EMCV genomic RNA equivalents per milligram.

### Antibiotic treatment, FMT, and bacterial colonization.

Mice were given an antibiotic cocktail composed of 10 mg each of ampicillin, neomycin, metronidazole, and vancomycin (167 mg/μl) daily for 5 days via oral gavage. After the fifth day of oral gavage, antibiotics were added to the drinking water at a concentration of 1 g/liter for ampicillin, neomycin, and metronidazole and 500 mg/liter for vancomycin. Fecal samples collected from microbiota-depleted mice at the 5th day posttreatment were homogenized, plated on BHI agar with 10% sheep blood, and cultured under anaerobic conditions at 37°C for 2 days followed by incubation under aerobic conditions at 37°C for 1 day to confirm efficient microbial depletion. Animals were maintained with Abx- or PBS-containing water for the duration of the experiment (6, 20).

For FMT experiments, 200 mg of pooled feces pellets were homogenized with sterile silica beads in 1.5 ml PBS at 45 Hz for 1 min and filtered with 70-μm strainers. Mice were administered Abx as described above, Abx administration was discontinued on day 6, and Abx-treated mice were subjected to gavage with 150 μl filtered stool homogenates individually (16). For bacterial colonization experiments, Abx-treated mice were subjected to gavage with 10^10^ CFU of *B. coccoides*, *A. muciniphila*, *C. butyricum*, or L. reuteri in 150 μl PBS on the 6th day after Abx oral administration. At 48 h after FMT or bacterial colonization (on day 8 after oral Abx gavage), stool samples were collected to determine the efficiency of colonization, and colonized mice were inoculated intraperitoneally with EMCV at various doses.

### IFA and viral signal quantification.

For immunofluorescence assay, brain tissues were collected from either mock-infected or infected mice at 3 or 5 dpi, fixed in 4% buffered paraformaldehyde, and embedded in paraffin. Deparaffinized brain sections were incubated with 10% normal goat serum for 30 min to block nonspecific binding, costained with anti-EMCV VP2 (1: 500) and anti-GFAP (1:1,200; Abcam) monoclonal antibodies, or costained with anti-VP2 and anti-IBA1 monoclonal antibodies (1:800; Invitrogen). Following primary antibody incubation at 4°C overnight, a Cy3-conjugated anti-mouse secondary antibody at a dilution of 1:100 (Santa Cruz Biotechnology, Santa Cruz, CA) was added to the sections and incubated for 30 min at room temperature. Stained sections were imaged with a Nikon Eclipse C1 upright fluorescent microscope. The number of antigen-positive cells from 10 fields of view (200×) was combined when a single section was observed and averaged over three coronal brain sections per mouse from six infected mice (36).

### Splenocyte, bone marrow-derived macrophage, and peripheral blood mononuclear cell generation and treatment.

Spleens were dissected, and single-cell suspensions of splenocytes were generated by grinding through a 70-μm strainer. Erythrocytes were lysed with red blood cell lysis buffer (RCLB; HyClone), and remaining cells were resuspended in PBS supplemented with 2% FBS and 1 mM EDTA.

Bone marrow-derived macrophages (BMDMs) were generated by isolation of bone marrow cells from mouse femurs and tibiae after sacrifice. Cells were cultured at 37°C in DMEM supplemented with 20% FBS and 30% supernatant of filtered L929 cells for 7 days prior to the experimental procedure. BMDMs were infected with EMCV at MOI 5 for 8 h prior to harvest for the relevant groups.

PBMC isolation was performed by using density gradient centrifugation with a PBMC isolation kit (TBD).

### IFN-β protein analysis.

IFN-β protein quantification was performed using a mouse IFN-β enzyme-linked immunosorbent assay (ELISA) kit (ABclonal) according to the manufacturer’s instructions.

### Cytokine expression analysis.

RNA was isolated from tissues or cells using TRIzol reagent (Invitrogen) per the manufacturer’s instructions. Gene expression levels of *Ifnb*, *Isg15*, *Isg56*, *Irf7*, *Irf9*, *Stat1*, *Stat2*, *Oas1a*, *Mx1*, and *Cxcl10* (primers used for the assay are listed in Table S1) were determined via qRT-PCR and normalized to GAPDH expression. Results are presented as fold changes of cytokine expression in infected mice relative to that in mock animals (2^−ΔΔ^*^CT^*).

10.1128/mBio.00366-21.1 TABLE S1 Primer sets used for specific cytokine genes detection by quantitative reverse transcription-PCR. Download Table S1, DOCX file, 0.02 MB. Copyright © 2021 Yang et al. 2021 Yang et al. https://creativecommons.org/licenses/by/4.0/ This content is distributed under the terms of the Creative Commons Attribution 4.0 International license.

### Mononuclear phagocyte depletion.

Mononuclear phagocyte depletion experiments were performed as previously described. Briefly, animals were injected with 250 μl per mouse of clodronate liposomes (Yeasen) or control liposomes intraperitoneally 2 days prior to Abx treatment, on the day of Abx treatment, and then on days 2 and 5 after Abx treatment.

### DNA extraction, 16S rRNA amplicon sequencing, and data analyses.

Genomic DNA of the fecal samples was extracted using the ALFA-SEQ Advanced stool DNA kit (Magen). The quality and quantity of DNA were measured using a NanoDrop One instrument (Thermo Fisher Scientific). Subsequently, bar-coded PCR primers targeting the V3-V4 region of bacterial 16S rRNA genes were used to generate amplicons (forward primer, ACTCCTACGGGAGGCAGCA; reverse primer, GGACTACHVGGGTWTCTAAT), and multiplex sequencing of amplicons with sample-specific barcodes was performed using an Illumina Novaseq 6000 platform (paired-end 2 × 250-nucleotide reads; Guangdong Magigene Biotechnology Co., Ltd., Guangzhou, China).

The raw sequencing data were filtered using fastp (version 0.14.1, https://github.com/OpenGene/fastp) with the parameters -W4 -M20 and further processed with cutadapt (https://github.com/marcelm/cutadapt/) to remove the primer sequences to obtain the paired-end clean data. Subsequently, the usearch -fastq_mergepairs tool (version 10; http://www.drive5.com/usearch/) was utilized to merge the raw tags, which were later trimmed by fastp to get the clean tags. The operational taxonomic units (OTUs) were then clustered with a cutoff value of 97% similarity using UPARSE software. Each representative sequence of these OTUs was assigned using the SILVA database to annotate taxonomic information. The richness of certain commensal bacteria taxa was calculated using the usearch -alpha_div (version 10; http://www.drive5.com/usearch/) according to the OTU abundance. Based on the relative abundance of species at each classification level in otu_table, R software was used to draw the histogram, heat map, and ternary-phase diagram.

### Flow cytometry and cell sorting.

Splenocytes and PBMCs were harvested to analyze levels of different antigen on the surfaces of different cell subsets following blockade of Fcγ receptors with anti-CD16/32 (eBioscience). Fluorescently conjugated antibodies used include those specific to CD3, CD4, CD8, CD19, CD11b, CD11c (BioLegend), Ly6c, F4/80, MHC-II, and PDCA-1 (eBioscience). Inflammatory monocytes were identified as Ly6c^+^ and CD11b^+^. PBMC and splenic mononuclear phagocytes were identified as F40/80 and CD11b double positive. NK cells were identified as CD3^−^ and NK1.1^+^. cDCs were identified as F4/80^−^, CD11c^+^, and MHC-II^hi^. pDCs were identified as CD11c^int^ and PDCA-1^+^. CD4 T cells, CD8 T cells, and B cells were identified as CD3^+^ CD4^+^, CD3^+^ CD8^+^, or CD19^+^ respectively. For intracellular staining, cells were permeabilized with Cytofix/Cytoperm buffer (BD) and stained for granzyme B. Splenic mononuclear phagocytes were sorted as F40/80 and CD11b double positive subsets by flow cytometry using the BD FACSAria II cell sorter.

### *In vitro* phosflow STAT1 staining of mononuclear phagocytes.

Splenic mononuclear phagocytes were stimulated with 200 ng/ml recombinant IFN-γ (R&D Systems) for 16 h. Medium was subsequently removed and replaced with 0.05% trypsin, and cells were incubated at 37°C for 2 min. Cells were then fixed with 4% paraformaldehyde (PFA) for 10 min, stained for surface markers, permeabilized with Cytofix/Cytoperm buffer, and stained for pSTAT1 with phycoerythrin (PE)-conjugated anti-STAT1 (pY701) antibody (BioLegend).

### Statistical analysis.

Statistical analyses were performed with Prism GraphPad software v 8.0. Error bars represent standard errors of the means in all figures and *P* values were determined by unpaired, two-tailed Student's *t* test. A log-rank test was used for survival curves.
