# Supplementary figures and images for: The Intestinal Microbiome Primes Host Innate Immunity against Enteric Virus Systemic Infection through Type I Interferon
Source: mBio. 2021 May 11;12(3):e00366-21. doi: 10.1128/mBio.00366-21 (PMC8262959; doi:10.1128/mBio.00366-21)

**A**

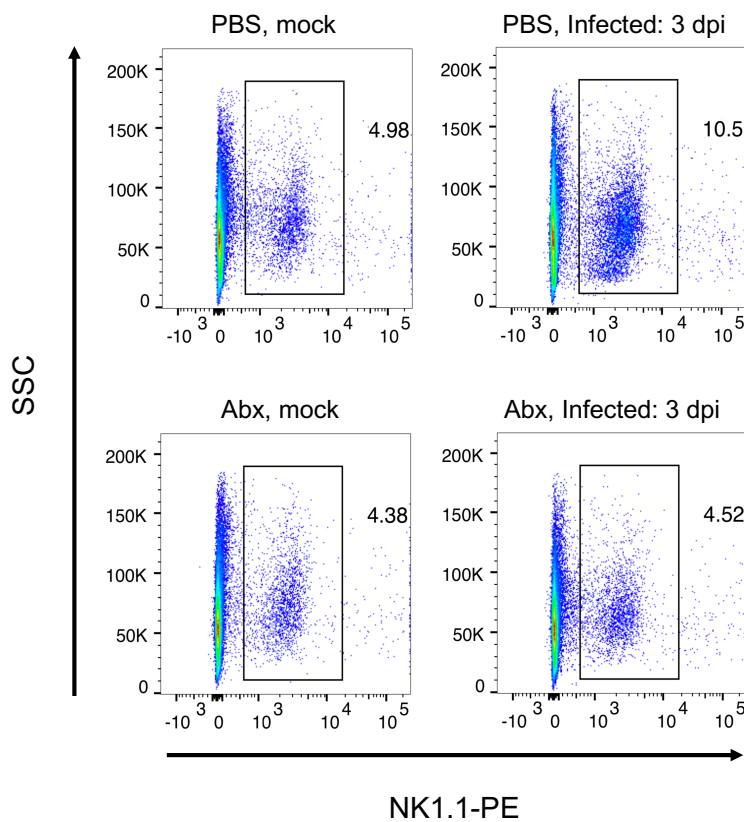

**B**

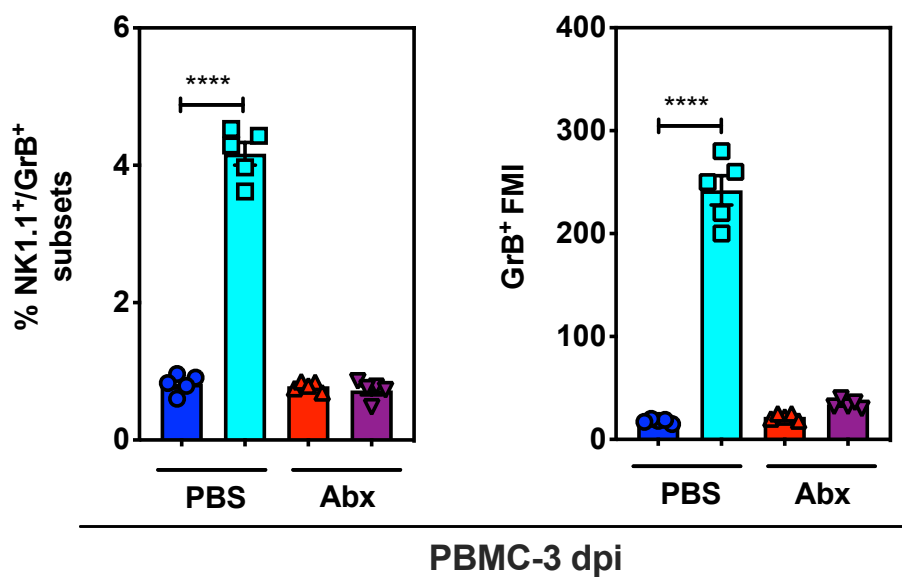

Supplement: FIG S1 [file mbio.00366-21-sf001.pdf]

**Fig. S2****A**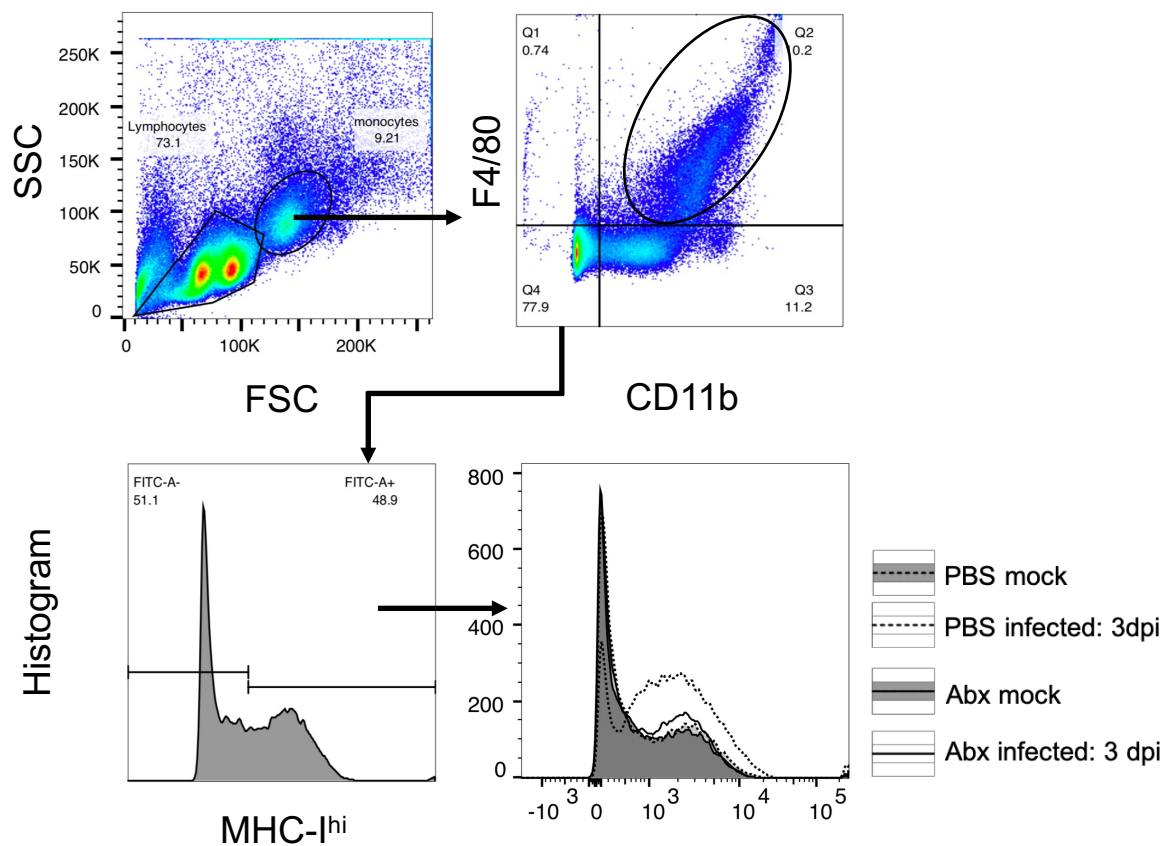**B**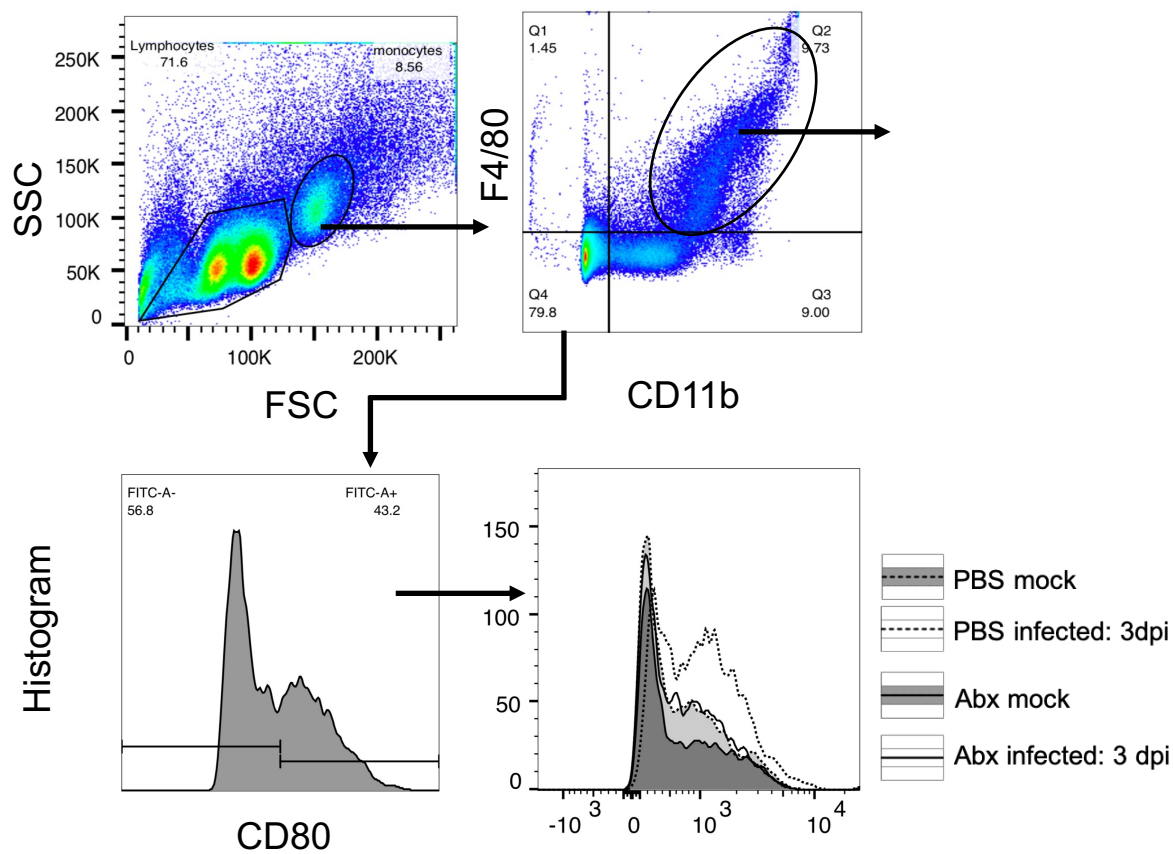

Supplement: FIG S2 [file mbio.00366-21-sf002.pdf]

**A****Control reagent**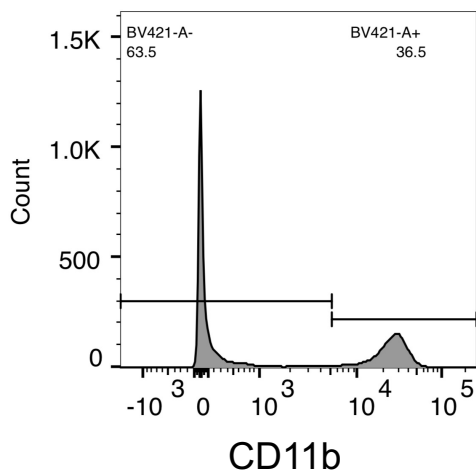**B****clodronate liposomes**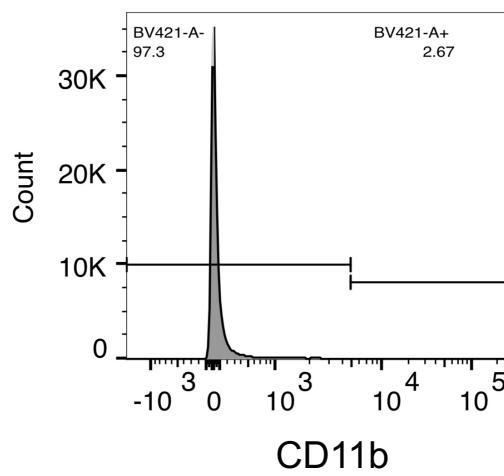

Supplement: FIG S3 [file mbio.00366-21-sf003.pdf]

**Fig. S4**

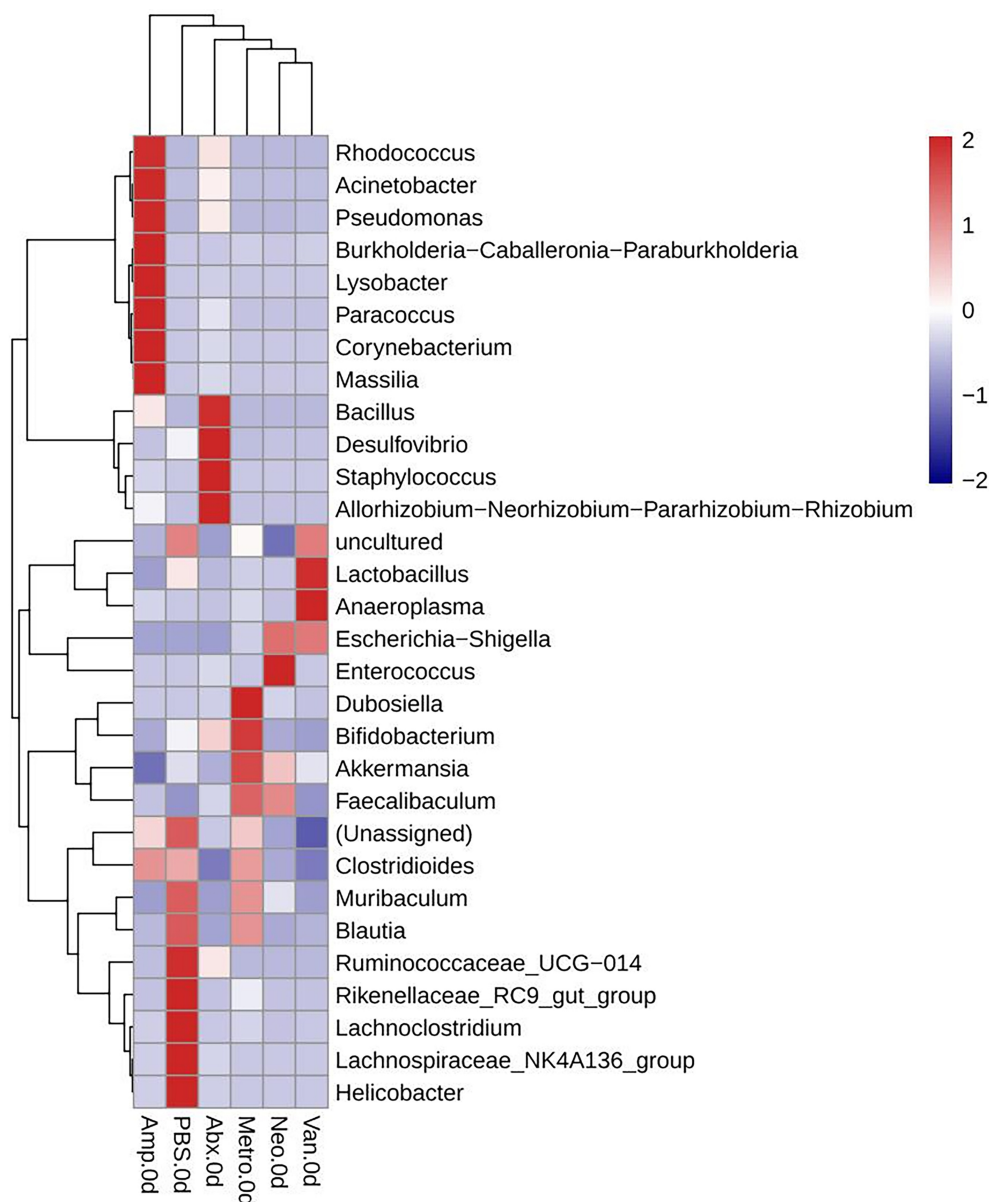

Supplement: FIG S4 [file mbio.00366-21-sf004.pdf]

Fig. S5

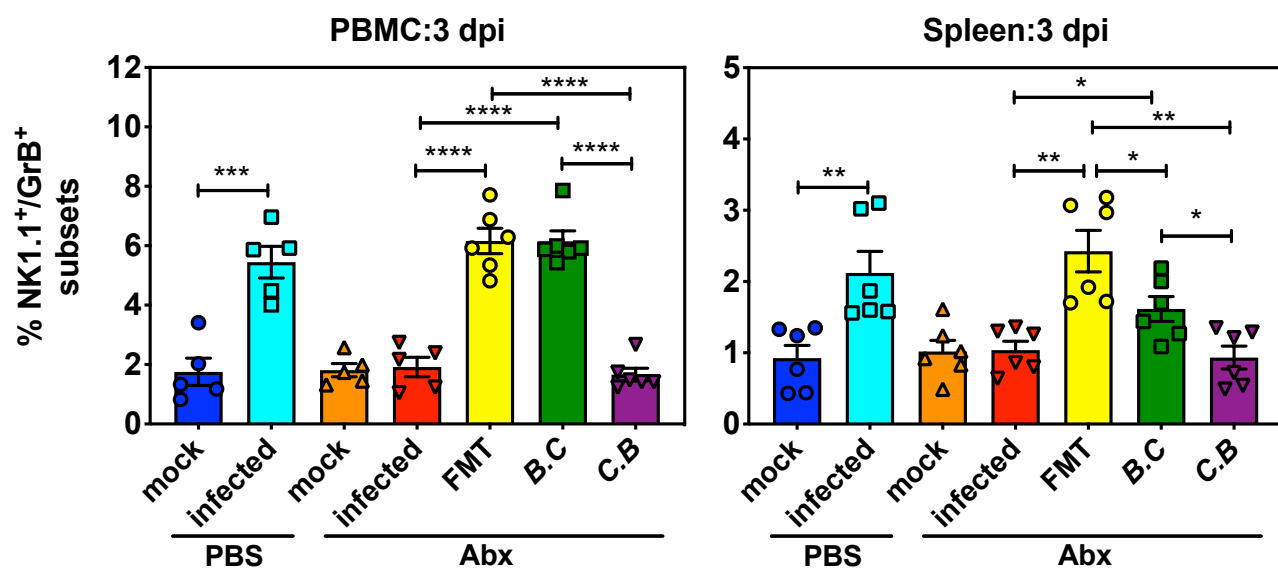

Supplement: FIG S5 [file mbio.00366-21-sf005.pdf]

**Fig. S7**

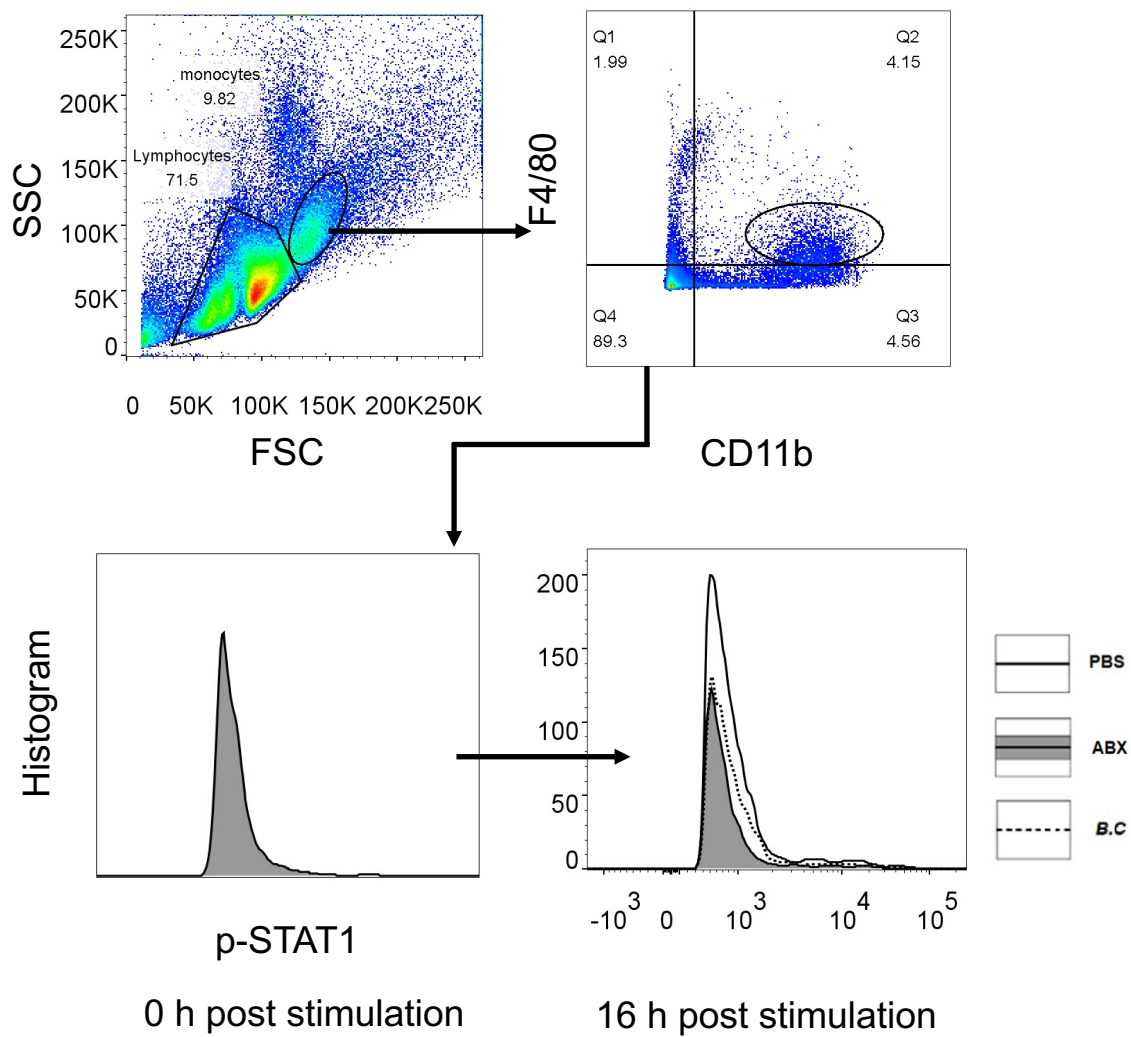

Supplement: FIG S7 [file mbio.00366-21-sf007.pdf]

Fig. S6

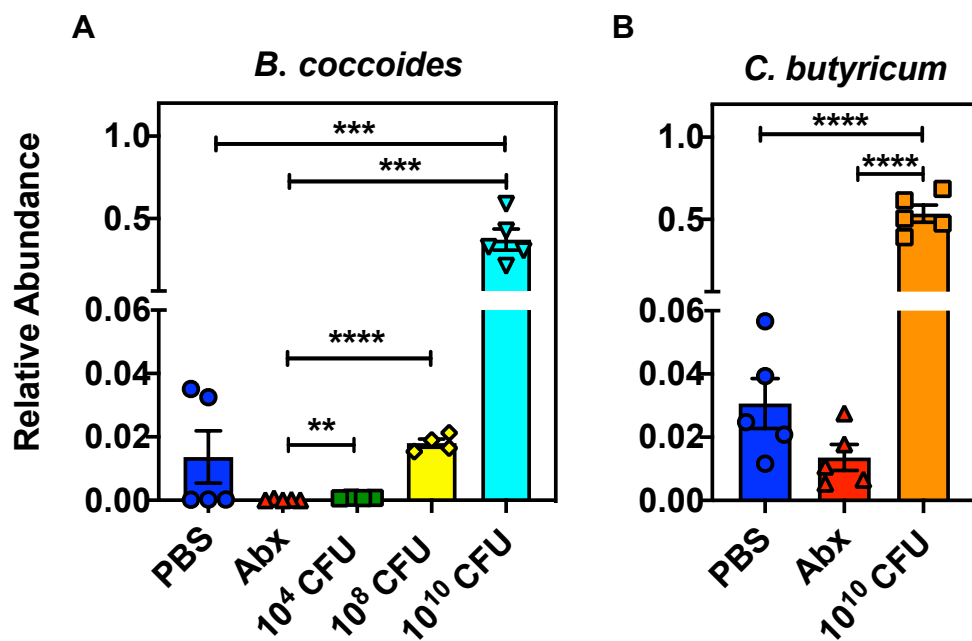

Supplement: FIG S6 [file mbio.00366-21-sf006.pdf]
