# Supplementary material for: The Intestinal Microbiome Primes Host Innate Immunity against Enteric Virus Systemic Infection through Type I Interferon
Source: mBio. 2021 May 11;12(3):e00366-21. doi: 10.1128/mBio.00366-21 (PMC8262959; doi:10.1128/mBio.00366-21)
Supplement: TABLE S1 [file mbio.00366-21-st001.docx]

Table S1. Primer sets used for quantitative reverse transcription-PCR

| Gene | Premier sequence |  |
| --- | --- | --- |
|  | Forward primer | Reverse primer |
| *Ifnb* | 5'-CGGACTTCAAGATCCCTATGGA-3’ | 5'-TGGCAAAGGCAGTGTAACTCTTC-3’ |
| *Isg15* | 5'- TTTTCAGCTCTGCATCGTTTTGGGT-3’ | 5'- CCTTGAAACAGCATCTGACTCCTT-3’ |
| *Isg56* | 5'-AACATGATCGTGCGCTCTGCAAGTGCAGC-3’ | 5'- AAGGAATAGTGCAGACAGGCAGGA-3’ |
| *Irf7* | 5'- CTTCAGCACTTTCTTCCGAGA-3’ | 5'- TGTAGTGTGGTGACCCTTGC-3’ |
| *Irf9* | 5'- ATTTCGGTCGTAGGGATCTGG-3’ | 5'- GCACAGCGGAAGTTGGTCT-3’ |
| *Stat1* | 5'- GACTACCACTGAGATGACCCAGC-3’ | 5'- ATTTCCTCCCCAAATGTTTTCA-3’ |
| *Stat2* | 5'- TCCTGCCAATGGACGTTCG-3’ | 5'- GTCCCACTGGTTCAGTTGGT-3’ |
| *Oas1a* | 5'- GGGAAGATAGCCGAAGACCT-3’ | 5'- CCTCGAGGGCTGTCAATCT-3’ |
| *Mx1* | 5'- AGGGGCCATCACATTCACAT-3’ | 5'- AGATACTTCAGGGGATTCTC-3’ |
| *Cxcl10* | 5'- TTTCTGCCTCATCCTGCTG-3’ | 5'- CTCATCATTCTTTTTCATCGTG-3’ |
| *Gapdh* | 5'- GCCTTCCGTGTTCCTACCC-3’ | 5'- CCCTCAGATGCCTGCTTCAC-3’ |
